# VH1-69 Utilizing Antibodies Are Capable of Mediating Non-neutralizing Fc-Mediated Effector Functions Against the Transmitted/Founder gp120

**DOI:** 10.3389/fimmu.2018.03163

**Published:** 2019-01-15

**Authors:** S. Abigail Smith, Samantha L. Burton, William Kilembe, Shabir Lakhi, Etienne Karita, Matt Price, Susan Allen, Cynthia A. Derdeyn

**Affiliations:** ^1^Yerkes National Primate Research Center, Emory University, Atlanta, GA, United States; ^2^Emory Vaccine Center, Emory University, Atlanta, GA, United States; ^3^Zambia Emory HIV Research Project, Lusaka, Zambia; ^4^Projet San Francisco, Kigali, Rwanda; ^5^International AIDS Vaccine Initiative, New York, NY, United States; ^6^Department of Epidemiology and Biostatistics, University of California, San Francisco, San Francisco, CA, United States; ^7^Department of Pathology and Laboratory Medicine, Emory University, Atlanta, GA, United States

**Keywords:** HIV-1, gp120, VH1-69, non-neutralizing Fc-mediated effector function, neutralization

## Abstract

Multiple antibody effector functions arise in HIV-1 infection that could be harnessed to protect against infection or clear the persistent reservoir. Here, we have investigated the genetic and functional memory B cell and antibody landscape present during early infection in six individuals infected with either subtype A, C, or an A/C recombinant HIV-1. These individuals demonstrated varying levels of plasma autologous neutralization (nAb) against the transmitted/founder envelope (T/F Env) pseudovirus and non-neutralizing Fc-mediated effector function (nnFc) antibody-dependent cell-mediated cytotoxicity (ADCC) against the T/F Env gp120 protein at ~7 months after infection. Genetic analysis of the immunoglobulin heavy (VH) and light (VL) chain variable domain gene segments from 352 autologous T/F Env gp120-specific single B cells recovered at this same 7-month time-point revealed an over-representation of the VH1-69 germline in five of six individuals. A defining feature of the VH1-69 utilizing gp120-specific antibodies was their significantly more hydrophobic complementarity-determining region-2 (CDRH2) regions compared to other VH CDRH2 sequences from each individual. While none of the VH1-69 antibodies possessed strong neutralizing activity against virions pseudotyped with the autologous T/F Env, almost a third were capable of mediating high ADCC activity, as assayed by intracellular granzyme B activity in CEM.NKr.CCR5 target cells coated with autologous T/F Env gp120. High ADCC mediating VH1-69 antibodies exhibited shorter complementarity-determining region-3 (CDRH3) lengths and a more neutral isoelectric point than antibodies lacking this function. In the individual that developed the highest autologous ADCC responses, the high granzyme B producing antibodies bound to surface expressed envelope in the absence of CD4 and were not enhanced by the addition of soluble CD4. Overall, VH1-69 utilizing antibodies are commonly induced against gp120 in diverse HIV-1 infections and a subset of these antibodies can mediate ADCC functions, serving as a bridge between the innate and adaptive immune response to HIV-1.

## Introduction

The potential antibody repertoire in humans is a stunning example of the creative capacity of the evolutionary process. Differential combinations of heavy-chain germline V, D, and J gene segments, kappa or lambda light-chain V and J combinations, all combined with the additional junctional diversity found within both the heavy and light-chain rearrangements, results an estimated 1 × 10^8^ to 1 × 10^15^ potential B cell receptor combinations (1, 2). This diversity is the foundation for B cell receptor repertoires in individuals that can interact, to some degree, with essentially any pathogen encountered over the course of a lifetime. From this starting point, reactive B cells evolve via somatic hypermutation and affinity maturation, generating antibodies with increasing affinity for specific epitopes associated with specific pathogens, and ultimately the ability to exert neutralizing and/or non-neutralizing effector functions, to not only clear current infections, but prevent or reduce the pathogenicity of future infections by the same (or similar) pathogen (3, 4). While this process has been capitalized on by numerous vaccination strategies to generate protective antibody responses in the absence of true primary infection, we have yet to generate a highly protective prophylactic vaccine to HIV-1. An HIV-1 vaccine will need to be given to a genetically diverse human population, and induce a protective immune response against HIV-1 variants of unprecedented genetic diversity (5, 6). However, the antibody response to HIV-1 infection is not necessarily consistent or predictable between individuals. Neutralizing (nAb) and non-neutralizing Fc-mediated (nnFc) activities against the autologous virus develop at different rates between individuals (7–13). Autologous nAb are initially strain-specific and target a single epitope that differs across individuals. In contrast, broadly neutralizing antibodies (bnAbs) are capable of neutralizing a large percentage of genetically diverse HIV-1 variants; however, they develop only after several years of infection in a small percentage of infected individuals, utilize a number of different heavy and light chain germline genes and combinations, and often exhibit exceptionally rare features (14–18). Nevertheless, bnAbs remain a major goal for some HIV vaccine development efforts. A hopeful strategy for the induction of bnAbs is to target germlines of interest and guide the evolution of these specific lineages along a pre-defined trajectory. This remains an uphill battle considering the randomness and diversity inherent in the normal human antibody selection and maturation process (19). Thus, anything resembling immunological convergence, especially within the context of HIV-1 infection, could be a source of cautious optimism.

In HIV-1 infection, nnFc activities such as antibody-dependent cell-mediated cytotoxicity (ADCC), have been associated with slower disease progression and elite control of viral replication, as well as reduced transmission in a number of studies in humans and non-human primates (20–27). Unfortunately, relatively little is known about the genetic and biochemical characteristics of antibodies mediating these responses during natural infection, or the defining features of antigens that are capable of inducing them. The immunoglobulin VH1 subgroup has been implicated in two analyses of vaccine-induced monoclonal antibodies (mAbs) capable of anti-HIV ADCC activity (28, 29). More specifically, VH1-69 germline derived antibodies are of interest, due to their propensity to combine a hydrophobic CDRH2 with an acidic CDRH3 that facilitates binding to CD4-induced (CD4i) epitopes on gp120, with the potential to mediate ADCC (30, 31). However, this has yet to be explored in depth within the context of mAb interactions with autologous T/F gp120 in early natural infection.

Here, we have capitalized on a unique opportunity to shed light on the B cell and antibody landscape in six individuals early in Subtype A (*n* = 2), Subtype C (*n* = 2), or Subtype A/C (*n* = 2) HIV-1 infection, where the development of neutralization breadth in plasma at 3-years post-infection was previously characterized (32). While autologous nAb and ADCC activity generally developed by 3 years post-infection in most individuals, marked diversity in both nAb and ADCC responses was observed between these individuals at ~7 months post-infection. An analysis of 352 memory B cells specific for the autologous T/F Env gp120 surface subunit isolated at this same time-point revealed the VH1-lineage was dominant, with VH1-69 heavy-chain germline genes over-represented in five of six individuals, regardless of subtype of the infecting variant or of individual host VH1-69 allelic variation. A subset (28%) of these dominant VH1-69 utilizing antibodies were capable of mediating high ADCC activity against CEM.NKr.CCR5 target cells coated with autologous T/F gp120. This subset displayed genetic features and epitope specificity distinct from the low ADCC VH1-69 mAbs. These results suggest in HIV-1 infection, VH1-69 utilizing mAbs with distinctive genetic features could be immunological “first-responders,” mediating ADCC effector function against autologous gp120 while nAb develops.

## Methods

### Ethics Statement

The participants, R774F, R53F, Z1800M, Z1047M, R1142F, and R66M, were among a group of individuals enrolled in heterosexual discordant couple cohorts that were selected for study based on rapid screening of adults with recent history of HIV exposure in Rwanda and Zambia. After obtaining written informed consent, blood samples were collected from participants longitudinally, every 1–3 months, depending on enrollment date. All couples in the cohort were provided monthly counseling and testing prior to the HIV-negative partner becoming positive. The procedures for written informed consent and research activities were approved by institutional review boards at all collaborating clinical research centers, with further compliance to human experimentation guidelines set forth by the United States Department of Health and Human Services. The study was reviewed and approved by the Republic of Rwanda National Ethics Committee, Emory University Institutional Review Board, and the University of Zambia Research Ethics Committee. To protect confidentiality, all subject identification numbers were anonymized by assigning a coded ID that removes any identifying information.

Blood was also obtained from normal, HIV-1 seronegative human volunteers at Emory University through an Institutional Review Board approved phlebotomy protocol. This protocol also anonymized volunteers by assigning coded IDs to remove any identifying information.

### Study Population

The subjects studied here were enrolled in Protocol C, a uniform vaccine-preparedness study developed and implemented by the International AIDS Vaccine Initiative (IAVI; http://www.iavi.org) that was carried out at multiple sites in Africa, including the Zambia-Emory HIV Research Project (ZEHRP, coded IDs Z1800M, Z1047M) and Projet San Francisco (coded IDs R774F, R53F, R1142F, R66M) (33, 34). Projet San Francisco subjects were associated with epidemiologically linked donors, while the two subjects from ZEHRP were not. These subjects were also included in our previous study (32).

### Cell Line Authentication

Genetica Cell Line Testing was used to authenticate 293 and TZM-bl cell lines. 293T cells: 88.9% identity, 80.00% match to HEK 293T/17. Expi293F: 90.32% identity, 93.33% match to HEK-293 [293] (ATCC CRL-1573). TZM-bl: 100.00% identity, 100% match to HeLa (ATCC CCL-2). All lines were mycoplasma negative. CEM.NKr.CCR5 cells were obtained through the NIH AIDS Reagent Program, Division of AIDS, NIAID, NIH: CEM.NKr.CCR5 Cells from Dr. Alexandra Trkola (35–37).

### Neutralization Assays

Generation of Env pseudoviruses and the experimental protocol for the TZM-bl neutralization assay have been described previously (9, 11, 38–49). As R66M began antiretroviral therapy at ~1.25 years post-infection, which can give a false impression of “neutralization” in the TZM-bl assay, IgG antibodies were purified from the 36.5-month plasma sample (Time Point 3) using a GE Healthcare Life Sciences Ab SpinTrap, according to the manufacturer's instructions (GE 28-4083-47). The concentration of the purified IgG was determined by ELISA and adjusted, and was then used in place of plasma in neutralization assays for this time point. In all other assays, plasma was used to evaluate polyclonal autologous neutralization. 2000 IU of each titered Env pseudovirus (in DMEM with ~3.5% (vol/vol) FBS (Hyclone, # SH30088.03) and 40 μg/mL DEAE-dextran) was mixed with five-fold serial dilutions (beginning at 1:100) of heat-inactivated plasma samples or purified polyclonal IgG and assayed for its inhibitory potential against the appropriate autologous Env pseudovirus using the TZM-bl indicator cell line, with luciferase as the readout. The average background luminescence from a series of uninfected wells was subtracted from each experimental well. All assays contained duplicate wells and were repeated at least once independently. For mAb neutralization, 2000 IU pseudovirus with 10 μg/ml of mAb and added to plated TZM-bl cells. Samples were run in triplicate, and when antibody yield was sufficient, this was duplicated. “Antibody only” wells were also included as a negative control to monitor for any potential cytotoxic activity of the mAb and/or elution buffer. HIV bnAb VRC01 was used as a positive control and anti-influenza HA mAb EM4C04 was used as a negative control, both at 10 μg/ml.

### Granzyme B Activity Assay

A panel of potential HIV-negative effector cell donors at Emory University were screened for CD16 158V/F polymorphisms via flow cytometry, similar to a method previously described (50). Donor PBMC were stained with APC conjugated anti-human CD56 (BioLegend, #318309), PacBlue anti-human CD3 (BioLegend, #300330), and either FITC anti-human CD16 (Thermo, #MHCD1601) for total CD16, or a FITC anti-human CD16 158V-specific clone MEM-154 (Thermo, #MAI-19563) and analyzed on an LSRII (BD Bioscience) instrument and with FlowJo 10.4 software (Treestar). The resulting ratio of the mean fluorescence of FITC-positive, CD56, CD3, cells (MEM-154:Total) was used to eliminate 158F homozygous donors, and identify 158V/F or 158V/V donors for downstream use in the GzB assay. Two donors were selected (Donor 190, Donor 457), and PBMC were isolated via Ficoll centrifugation and cryopreserved in liquid nitrogen (90% FBS, 10% DMSO) until needed.

Assessment of GzB activity was performed as previously described (51). T/F Env gp120 proteins with a C-terminal 6XHis-tag were synthesized by GENEART (ThermoFisher). Genes were synthesized and inserted into pcDNA3.3 and transfected into FreeStyle™ 293 cells. Proteins were purified via 5 ml HisTrap™ FF column, linear gradient from 20 to 500 mM Imidazole in PBS, 500 mM NaCl at 6 days post-transfection, with a HiLoad 26/600 Superdex200 polishing step. Cryopreserved PBMC from uninfected donors were recovered in RPMI (10% FBS (Hyclone) Pen/strep/l-glut) the night before the assay. To ensure consistency across assays, effector cell viability was required to be >80% on the day of the assay to proceed. CEM.NKr.CCR5 cells were coated with 8 μg gp120/million cells at RT for 45 min. Dyes TFL4 and NFL1 were added according to the manufacturer's instructions for 15 min in a 37 degree C water bath (OncoImmunin, Inc. GranToxiLux PLUS! and NFL1). CEM.NKr.CCR5 target cells were washed 2x in complete RPMI media, then mixed with primary donor PBMC cells at a 30:1 Effector:Target ratio (25 μl 300,000 Effector cells; 25 μl 10,000 Target cells), along with 75 μl of GzB substrate, and incubated for 5 min at RT. Twenty five microliters of five-fold serially diluted plasma (beginning at a dilution of 1:200), 50 μg/ml HIV-Ig [NIH AIDS Reagent Program, Division of AIDS, NIAID, NIH: from NABI and NHLBI (cat# 3957) (52)] positive control, or 50 μg/ml commercial IgG (SouthernBiotech, #0150-01) negative control were added, and incubated at RT for 15 min. Tubes were spun at 300 × *g* for 1 min, and incubated at 37°C, 5% CO2, for 1 h. Fixed cells (final concentration 1.85% formaldehyde, 10 min) were washed, then resuspended in buffer for flow cytometric analysis. Because fixation could result in loss of GzB signal, samples were immediately analyzed on an LSRII (BD Bioscience) instrument and with FlowJo 10.4 software (Treestar). Target cell only controls were run to ensure proper gating of targets vs. effector cells. Target with added effector cells in the absence of antibody (media only) were included to quantify background GzB signaling. Values that became negative after subtraction of background were normalized to 0%. Negative (commercial IgG) and positive (HIV-Ig) controls were included in every assay run. Assays were not valid unless the percentage of GzB positive cells in the positive control tube were >10%, after subtraction of background activity. Only results from valid assays are included. Example Granzyme B assay flow-plots are included in Supplementary Figure 1. To test for inherent differences across the gp120 proteins that could influence the assay, negative and positive control GzB activity was examined. There were no statistical differences between the T/F gp120 proteins in background (Target + Effector cell only samples), negative control (commercial IgG), or positive control (HIV-Ig) GzB activity (Supplementary Figures 2A–C). There was also no statistical difference in all experimental values between the two effector cell donors, as values obtained from each donor were not statistically different (Supplementary Figure 2D, Mann–whitney, *p* > 0.05) and were highly correlated (Supplementary Figure 2E, Spearman, *p* < 0.0001, *r* = 0.83). To measure GzB activity induced by mAbs, the protocol was repeated in an identical manner, with all corresponding positive and negative controls, but with experimental mAbs tested at five-fold serial dilutions beginning at 5 μg/ml.

### B Cell Sorting

The same preparations of T/F Env gp120 proteins as used in the GzB assay, described in detail above, were used for single B cell sorting. Cryopreserved PBMC obtained from the sites in Rwanda and Zambia (~5–10 million cells/vial) were washed and resuspended in PBS + 2% FBS. After counting, cells were incubated with 8 μg of the autologous T/F gp120 per million cells and stained with: live/dead Aqua (Invitrogen, # L34957), anti-CD3 Pacific Blue (BD Pharmingen, # 558124), anti-CD19 BV650 (BioLegend, # 302237), anti-IgG FITC (BD Pharmingen, #555786), anti-His PE (Miltenyi Biotec, # 130-098-810), and single-cell sorted into 96-well plates containing 20 μl cell lysis buffer (Superscript III RT buffer, Tween, DTT, Invitrogen, #18080-044) (RNaseOUT, Invitrogen, #100000840). Sorts were carried out using a FACS Aria Cell Sorter and the following gating strategy: size, singlets, live, CD3-, CD19+, IgG+, gp120/His+. CD3+ gp120/His+ cells were monitored as positive controls for gp120 binding and detection.

Immunoglobulin heavy and light chain variable domain regions were PCR-amplified as previously described, using oligo-dT (ThermoScientific, #AM5730G) and SuperScriptIII (Invitrogen, #18080-044) (53). IgG-specific primer was also included in the RT reaction for individuals R53F, R774F, and Z1047M to increase recovery of heavy chain regions. Five microliters of the RT reaction was used to amplify heavy (IgG only), kappa, and lambda chain variable regions using high performance liquid chromatography (HPLC) purified primers as described in Liao et al. (53) and Phusion Hotstart II High Fidelity DNA Polymerase (Thermo Scientific, #F537S) for first round nested PCR. For the second round PCR reaction, 2.5 μl of first-round was used as a template. PCR amplified variable regions were gel purified (Qiagen, #28706) and combined with a CMV promoter containing DNA fragment, and the appropriate corresponding constant region DNA fragment (including a polyA tail) via overlapping PCR (53). Plasmids HV0024, HV0023, HV0025, and HV0026 were kindly provided by Dr. Larry (Huaxin) Liao at the Duke Human Vaccine Institute. The assembled full-length heavy- and light-chain segments were then cloned into pCR2.1TOPO-TA (ThermoFisher, # K4500-40) for long-term storage and mAb expression. VH and VL plasmid pairs were co-transfected at a 1:2 ratio into 6-well plates containing Expi293F cells (Thermo Fisher Scientific, Expi293 Expression System Kit, #A14635). Five to seven days post-transfection, mAbs were purified from the cell culture supernatant using Ab SpinTrap with Protein G Sepharose High Performance (GE Healthcare, # 28408347). The concentrations of the purified mAbs were quantified on an Octet RED96 using Anti-Human IgG Fc AHQ biosensors (ForteBio, #18-5001). mAb expression plasmids that could be stably amplified and resulted in transfections with yields >100 μg/ml were carried forward with antibody effector function characterizations.

### PCR and Sequence Analysis of Immunoglobulin Variable Domains

The nucleotide sequence of each PCR amplicon was determined by Beckman Coulter Genomics or Sequetech using the following primers: H-R474 (5′- GCTGTGCCCCCAGAGGTG-3′) or K-R405 (5′-GACAGATGGTGCAGCCACAGTTCG-3′) or L-R400 (5′-CAGAGTGACCGAGGGGGCAGC-3′). These partial sequences were analyzed with NCBI IGBLAST for putative germline gene identification (https://www.ncbi.nlm.nih.gov/igblast/igblast.cgi) (Supplementary Table 1) (54). After assembly of the PCR fragments and cloning, plasmids were sequenced with the appropriate reverse primer, as well as CL-F681 (5′- TCTGGGTTCCAGGTTCCACTGGTGAC-3′). The resulting sequences were re-analyzed using IMGT vquest (http://imgt.org/IMGT_vquest/vquest) for germline gene verification, framework and CDR mapping, quantification of percent identity to germline, CDR H3 and L3 amino acid length, molecular mass, and pI (Supplementary Table 1) (55, 56). Circos diagrams were generated via the Circos online tool (http://mkweb.bcgsc.ca/tableviewer/) (57). CDRH2 grand average of hydropathy (GRAVY) scores were calculated using an online tool (http://www.gravy-calculator.de/). CDRH3 tyrosine sulfation sites were enumerated with the Sulfinator online tool (http://web.expasy.org/sulfinator/) (58). Sequences were uploaded to Genbank under the following accession numbers: MK269362–MK270058.

### Assessment of Affinity and Epitope Binning Using Biolayer Interferometry (BLI)

Affinity assays were performed on an Octet RED96 (ForteBio, Inc, Menlo Park, CA) at 30°C, with 1,000 rpm agitation. Each mAb was immobilized on an Anti-hIgG Fc Capture (AHC) biosensor (Fortebio, # 18-5060) at a concentration of 25 μg/ml for 300 s, and a baseline reading was recorded for 60 s in kinetics buffer (PBS with 0.01% BSA, 0.02% Tween20, and 0.005% sodium azide). Sensors were then immersed in varying molarities of the corresponding autologous T/F gp120 protein for 300 s, then allowed to dissociate in kinetics buffer for 600 s. Sensors were regenerated before each kinetics assay. Human IgG at 25 μg/ml dipped into kinetics buffer was included on every assay plate for reference during analysis (SouthernBiotech, #0150-01). Molarity (range 5–0.06 μM), association time (range 300–600 s), and dissociation time (range 600–1,200 s) were varied as necessary to obtain appropriate X^2^ and *R*^2^ values. Anti-influenza HA antibody EM4C04 (kindly provided by Dr. Jens Wrammert, Emory University) and/or commercial IgG were included to survey for non-specific mAb binding against each gp120.

K_D_ values were calculated using ForteBio Data Analysis 9.0. After reference subtraction, Y-axis data was aligned to the 50–59.9 s of the baseline reading. For inter-step correction, data was aligned to the dissociation step. Data was then processed with Savitzky-Gola filtering, and curve fitting was performed using a 1:1 binding model. K_D_ values were averaged from ≥2 reads (average number of reads ≥3) with appropriate X^2^ and *R*^2^ (median X^2^ = 0.11, range = 0.00–1.94; median *R*^2^ = 0.99, range = 0.93–1.0) (Supplementary Table 3).

Binning (competition) assays were performed at 30 degrees C, with 1,000 rpm agitation. Synthesized gp120 (R66M T/F Env O20) at a concentration of 25 μg/ml was immobilized on an Anti-Penta-HIS (HIS1K, ForteBio, # 18-5120) biosensor for 300 s, and a baseline reading was recorded for 30 s in kinetics buffer. Sensors were then immersed in 0.66 μM primary (R66M-derived) mAb for 10 min. R66M-derived mAbs that resulted in a primary signal of <0.1 at 0.66 uM (lower affinity mAbs) were increased in molarity, as production allowed, to reach that threshold. mAbs that could not be produced at this concentration were not analyzed. After a second baseline reading for 30 s in kinetics buffer, sensors were immersed in 0.66 μM secondary antibody (“reference mAb”—VRC01, 3074, PGT121, R66M 7C12) (VRC01 mAb Heavy Chain Expression Vector and VRC01 mAb Light Chain Expression Vector was obtained through the NIH AIDS Reagent Program, Division of AIDS, NIAID, NIH: from Dr. John Mascola (cat# 12035 and 12036) (59, 60); 3074, kindly provided by Xiangpeng Kong, NYU School of Medicine; PGT121, PGT 121 was obtained through the NIH AIDS Reagent Program, Division of AIDS, NIAID, NIH: Anti-HIV-1 gp120 Monoclonal (PGT121) catalog #12343 (61) to assess reference mAb binding capacity in the presence of primary R66M mAb binding. Reference mAb as primary and secondary mAb was included as a positive control for competition (reduced reference binding). EM4C04 (anti-influenza mAb) primary and reference mAb secondary was used as an additional control for non-specific competition (peak reference binding). Binning values were calculated using ForteBio Data Analysis 9.0. R66M mAbs capable of reducing reference mAb binding to <50% of reference binding in the presence of EM4C04 primary (set at 100% binding), were considered competitors for that reference mAb.

### Flow Cytometric Analysis of mAb Binding to Surface Env With and Without sCD4

293T cells were transfected with a plasmid encoding the T/F *env* gene isolated from R66M (the same plasmid utilized for generation of R66M Env pseudovirus) using Fugene HD (Promega). At 48-h after transfection, cells were washed with PBS, and briefly trypsinized. After washing again with PBS, cells were aliquoted and resuspended in flow buffer (PBS + 5% FBS), with or without 25 μg/ml recombinant human sCD4 (R&D Systems, 514-CD), and allowed to incubate for 30 min at RT. Cells were then incubated with 25 μg/ml polyclonal IgG (SouthernBiotech, #0150-01), 5 μg/ml PGT121, 10 μg/ml of CD4i mAb17b, or an R66M mAb at RT for 30 min. After washing with flow buffer, samples were then incubated with Goat Anti-Human IgG Fc biotin (SouthernBiotech, # 2014-08) at 1:200 for 20 min at RT. After washing with flow buffer, samples were incubated with 1:1,000 PE-conjugated streptavidin (BD Pharmingen, #554061) for 20 min at RT, while protected from light. Cells were then washed and fixed for 10 min (3.7% formaldehyde). The percentage of PE/Env positive cells were determined via quantification on an LSRII (BD Bioscience) instrument and with FlowJo 10.4 software (Treestar). All samples were analyzed on at least three independent occasions. PGT 121 was obtained through the NIH AIDS Reagent Program, Division of AIDS, NIAID, NIH: Anti-HIV-1 gp120 Monoclonal (PGT121) (61). 17b was obtained through the NIH AIDS Reagent Program, Division of AIDS, NIAID, NIH: Anti-HIV-1 gp120 Monoclonal (17b) from Dr. James E. Robinson (62–67).

### Statistical Analyses

All graphing, calculations, and analyses were performed in Prism 6.0. Non-parametric statistical tests were used. A Mann–Whitney test was used to compare two groups; a Kruskal–Wallis test was used to compare more than two groups. Ordinary one-way ANOVA, Tukey's multiple comparison test was used for multiple comparisons. Chi-square test was used for contingency analyses. Spearman rank test was used to assess correlations.

## Results

### Marked Variation in the Development of Plasma Autologous nAb and Non-neutralizing Fc Activity During Early HIV-1 Infection

In a previous study, 21 HIV-1 infected individuals from serodiscordant couple cohorts in Zambia (Z) and Rwanda (R) were evaluated for viral and host factors that contributed to the development of plasma nAb breadth at 3-years after infection (32). Here, six of those subjects were included in a more focused investigation of autologous nAb and ADCC activity against the respective T/F Env. These six individuals were selected because they were infected by the types of HIV-1 variants that predominate in these cohorts, subtype A (R774F and R53F), subtype C (Z1800M and Z1047M) and A/C recombinant HIV-1 (R1142F and R66M), and because sample quality, quantity, and availability was suitable for the study, which included sorting of antigen-specific B cells from cryopreserved PBMC. Plasma samples used in the analyses were collected at Time Point 1 (average of 0.89 months post-antigen-positive test date, range 0–2.9 months), Time Point 2 (average of 7.5 months post-infection, range 5.1–8.7 months), and Time Point 3 (average of 37.5 months post-infection, range 36.4–40.2 months). The same plasma sample was used to measure both nAb and ADCC activity in each case. The T/F Env for each individual had been derived previously using reverse transcription and single genome PCR amplification of plasma collected at an average of 34 days after the estimated date of infection (range 22–65 days), as described in Smith et al. (32). Sequence analysis was used to identify an amplicon representing the T/F Env consensus sequence, which was cloned into an expression plasmid for generation of pseudovirus for use in the neutralization assay (32). The T/F Env gp120 coding sequence of each individual was used to manufacture the protein for use in the ADCC activity assay and subsequent single B cell sorting.

**Figure 1:**
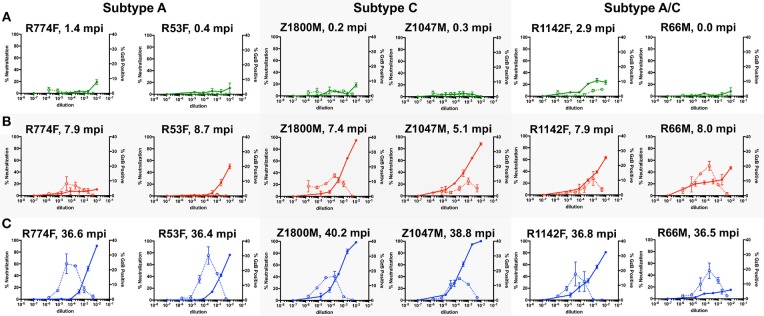
Evaluation of longitudinal nAb and ADCC capacity of plasma samples against the autologous T/F Env. nAb and ADCC activity in plasma collected at chronologic time points 1 (**A**, 0.87 months post infection), 2 (**B**, 7.5 months), and 3 (**C**, 37.5 months); All graphs show the percent neutralization of autologous T/F Env pseudovirus (closed symbols with solid line, left Y-axis) and percentage of fluorogenic GzB substrate positive CEM.NKr.CCR5 cells coated with autologous T/F Env gp120 (open symbols with dashed line, right Y-axis). The subjects are grouped by HIV-1 subtype, which is labeled on the top. The time points shown are 0 to 2.9 months after the first antigen-positive test (green), 5.1–8.7 months after the first antigen positive test (red lines), and 36.4–40.2 months after the first antigen positive test (blue lines). The X-axis indicates the reciprocal dilution of the individual's plasma plotted on a log_10_ scale. Symbols and error bars represent the mean and standard error of mean, respectively, between at least two replicate experiments for neutralization, and the mean and standard error of mean of independent experiments utilizing cells from two different donors for the GzB assay. Control data for (i) plasma nAb against a negative control VSV-g Env pseudovirus and (ii) GzB mediated by normal human serum against the T/F Env gp120 can be found in Supplementary Figure 2F.

Figure 1 shows the combined nAb and ADCC activity for the three longitudinal plasma samples against the autologous T/F Env for the six subjects. At Time Point 1 (Figure 1A, 0.89 months), three of the six individuals exhibited clear nAb activity that reached ~20% at the lowest dilutions (green, solid lines, left Y-axis). The highest levels of nAb were associated with individuals whose samples were obtained further from transmission (R774F, 1.4 months and R1142F, 2.9 months). ADCC activity was negligible in these early plasma samples, except for R1142F, which reached 4.6% GzB positive cells (green, dashed lines, right Y-axis). Thus, both nAb and ADCC antibody functions can be detected using the autologous T/F Env as early as 2.9 months after infection.

At Time Point 2 (Figure 1B, 7.5 months), nAb activity against the T/F Env exceeded 50% in five of six individuals at a 1:100 dilution of plasma, and was more than 80% in two individuals (red, solid lines, left Y-Axis). The exception was R774F, whose plasma nAb activity had not increased from that present at Time Point 1. ADCC activity was also readily detectable in five of six individuals at Time Point 2, with the average peak percentage of GzB positive target cells ranging from 8.1 to 20.2% at various plasma dilutions. In contrast, ADCC activity in R53F plasma remained indistinguishable from background, even though 50% neutralization of the T/F Env was achieved at this time point, suggesting that these two antibody populations were distinct.

At Time Point 3 (Figure 1C, 37.5 months), very potent autologous nAb was present in five of six individuals, exceeding 80% neutralization at the lower plasma dilutions, with two individuals exhibiting complete neutralization of the T/F Env (blue, solid lines, left Y-axis). nAb in R66M plasma was notably low at this later time point. High ADCC responses, peaking at 14.7–30.2% positive for GzB at various plasma dilutions, were present in all six individuals. Of note, when this data is compared with previously reported neutralization breadth at 3-years post-infection for this small set of individuals, no association between autologous ADCC activity and the development of neutralization breadth is apparent (18, 32). Overall, autologous nAb and ADCC activity developed in all six subjects to varying degrees, but the individual trajectory and magnitude varied.

### Memory B Cells With VH1-69 Dominate the Early Anti-gp120 Response in Five of Six Individuals

**Figure 2:**
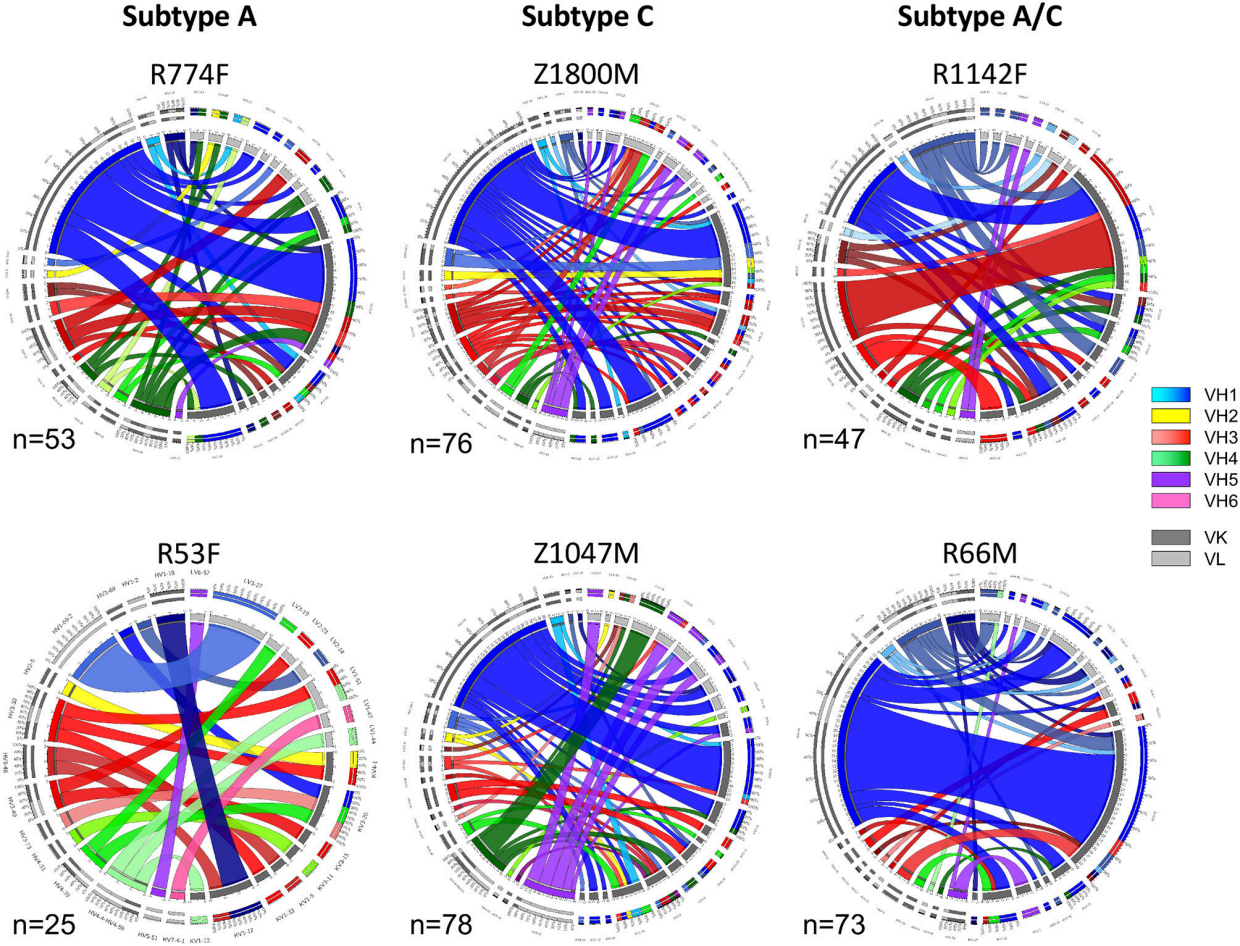
Circos diagrams of VH and VL pairing networks at 7.5-months post-infection (time point 2). Germline genes were assigned to each VH and VL sequence from each gp120-reactive single B cell using NCBI IGBLAST and vquest analysis. Circos diagrams were utilized to illustrate the network of VH germline (shades of blue VH-1, yellow VH-2, shades of red VH-3, shades of green VH-4, purple VH-5, pink VH-6) with the paired VL κ (dark gray) or λ (light gray) germline from individuals infected by HIV-1 Subtype A (left column), Subtype C (center column), and Subtype A/C unique recombinants (right column). Band width is proportional to the number of paired sequences that contain those VH and VL pairs. The total number of paired sequences analyzed for each individual is shown.

To understand the early antibody responses to the autologous HIV-1 T/F Env gp120 at higher resolution, variable heavy (VH) and variable light (VL) gene segments were recovered from single IgG+ memory B cells sorted for reactivity with the autologous T/F Env gp120 protein using cryopreserved PBMC collected at 7.5 months after infection (Time Point 2). This approach was designed to capture a wide variety of Env-specific B cells that were activated and selected for survival during the first 7 months of HIV-1 infection, and had the capacity to contribute to the plasma nAb and ADCC functions observed at this time point (Figure 1B). A total of 352 Env-specific memory B cells were recovered, and the VH and VL germline gene usage and pairings present in each subject are illustrated in Figure 2. In the Circos plots, each colored ribbon indicates a unique VH/VL combination, and the width is proportional to frequency (57). Broad diversity of VH and VL usage and pairing was observed in each individual, supporting that a highly polyclonal antibody response against epitopes in gp120 had occurred during the first months of infection.

**Figure 3:**
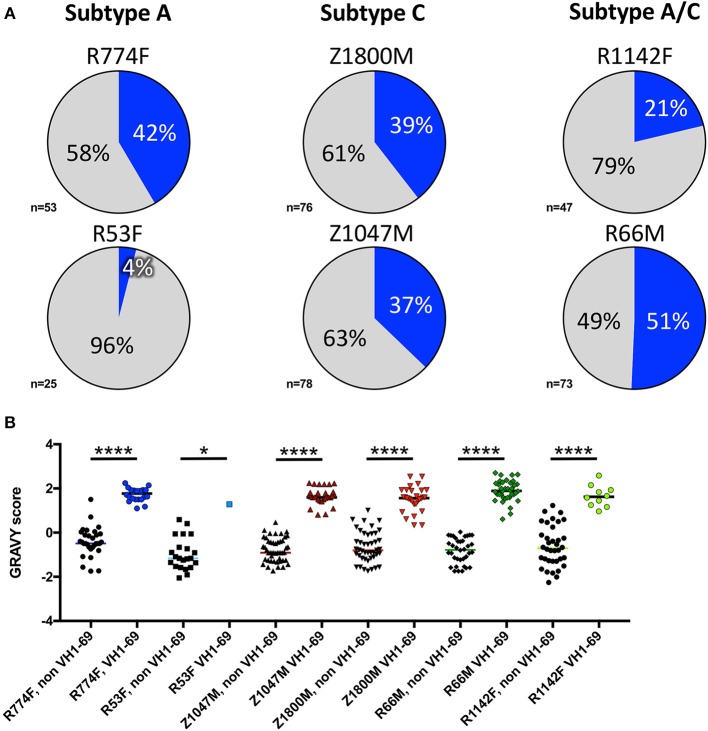
VH1-69 is over-represented among anti-gp120 memory B cells at 7.5-months post-infection. **(A)** Percentages of VH1-69 germline usage (blue graph sections) vs. all other variable heavy-chain germline genes (gray graph sections) isolated from memory B cells reactive to autologous T/F gp120 in six individuals infected with Subtype A (left column), Subtype C (center column), and Subtype A/C unique recombinants (right column). The total number of heavy chain sequences analyzed for each individual is shown. **(B)** To determine whether these VH1-69 sequences maintained a hydrophobic complementarity-determining region-2 (CDRH2) associated with VH1-69, grand average hydropathy (GRAVY) scores (Y-axis) were calculated from non-VH1-69 (black symbols) and VH1-69 sequences (colored symbols) obtained from each individual (http://www.gravy-calculator.de), bars represent median values. Individual codes are listed below the X-axis. VH1-69 CDRH2 were significantly more hydrophobic (positive values) than the non-VH1-69 CDRH2 in every individual (Ordinary one-way ANOVA, Tukey's multiple comparisons test: R53F *p* = 0.018, all others *p* < 0.0001). Raw values are reported in Supplementary Table 1.

Notably, VH1-derived B cells were commonly elicited in these individuals (Figure 2, shades of blue), similar to what has been described in chronic HIV-1 infection, VH1-derived B cells (68–72). Specifically, VH1-69 was the dominant VH germline gene utilized by T/F gp120-specific memory B-cells in five of the six individuals studied (Figure 2, royal blue). The frequency of VH1-69 B cells in those five individuals ranged from 21 to 51% (Figure 3A). Indeed, the VH1-69-lineage was not only dominant, but also over-utilized in all five individuals when compared to frequencies obtained from circulating B cells with successfully rearranged IGHV genes [reported range 3.1–9.7 73], or a previous analysis of eight HIV-1 vaccine volunteers pre-vaccination [reported range 0.7–8.2% IgG peripheral blood 74]. Therefore, we investigated in more detail the dominance of VH1-69 to understand the impact of this immunological phenomenon on the early antibody responses.

Pathogenically cross-reactive VH1-69 lineages likewise dominated a DNA prime/trivalent gp140 protein boost HIV vaccination regimen (74). And, hydrophobic interactions between VH1-69 CDRH2 and hydrophobic cavities within HIV-1 gp120 have also previously been observed (30). In the data collected here, grand average hydropathy (GRAVY) scores of isolated T/F gp120-specific VH1-69 CDHR2 amino acid sequences ranged from 0.34 to 2.7, with median GRAVY scores ranging from 1.28 to 1.89, indicating high hydrophobicity (Figure 3B, Supplementary Table 1). In contrast, the median CDRH2 GRAVY score for the corresponding non-VH1-69 T/F gp120-specific B cells for each individual was significantly lower, ranging from −1.16 to −0.46 (Ordinary one-way ANOVA, Tukey's multiple comparisons test: R53F *p* = 0.018, all others *p* < 0.0001). These observations reinforce the concept that high CDRH2 hydrophobicity is a unique and universal feature of the VH1-69 antibodies.

**Figure 4:**
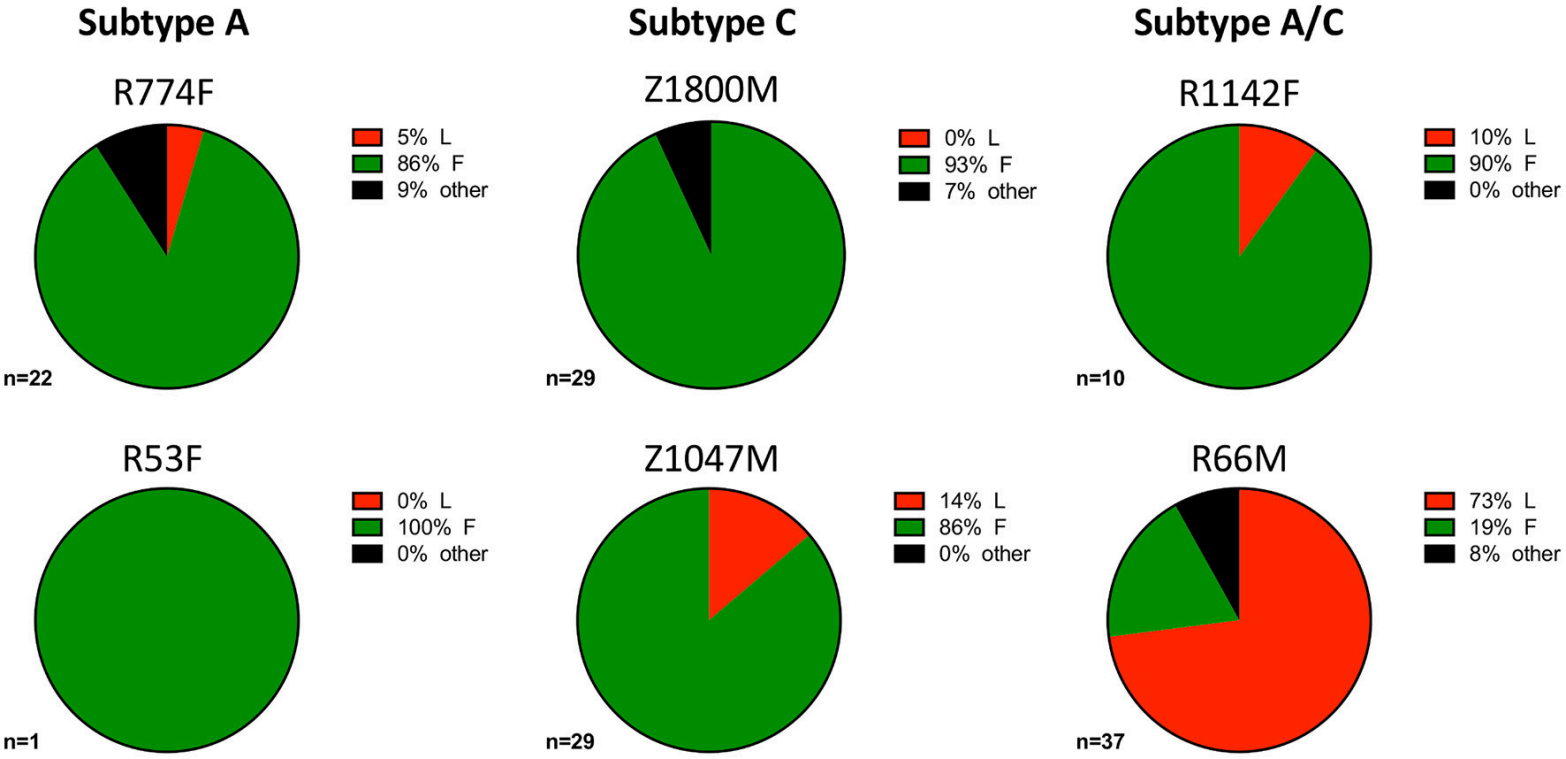
VH1-69 polymorphisms at amino acid 54 within complementarity-determining region-2 (CDRH2). The frequency of 51p1-like 54F (green), hv1263-like 54L (red), and other amino acids in six individuals infected with Subtype A (left column), Subtype C (center column), and Subtype A/C unique recombinants (right column) is illustrated. The 54F polymorphism was dominant except for R66M (bottom right), where 54L was present in the majority of the anti-gp120 VH1-69 sequences and R53F where only one VH1-69 sequence was present. A minority of VH1-69 sequences contained “other” amino acids (black) at this position in three individuals: R774F, Z1800M, and R66M. The total number of VH1-69 sequences analyzed for each individual is shown. Raw values are reported in Supplementary Table 2.

During influenza infection, the presence of a phenylalanine (F) or leucine (L) polymorphism at position 54 can strongly influence the frequency of VH1-69 utilization in anti-influenza antibodies, and their resulting functions (75). Though individuals' genomes were not sequenced to determine specific alleles within this study, this information was inferred from the isolated VH1-69 sequences. Within this group of individuals, VH1-69 containing 54F (associated with neutralization of influenza) comprised the majority of anti-gp120 VH1-69 sequences in five of the six individuals (Figure 4, green). These findings reflect previous analyses that noted the 54L-homozygous genotype is rarely found in African populations, with 54F-homozygous and 54F/L-heterozygous alleles composing the vast majority of VH1-69 genotypes (2, 76). Interestingly, the 54L polymorphism was observed in the majority of VH1-69 sequences from one individual, R66M (73%, Figure 4, Supplementary Tables 1, 2), while it was at a much lower frequency of between 0 and 14% in the other individuals. VH1-69 sequences that contained an amino acid other than F or L at position 54 were isolated from three individuals: R774F, Z1800M, and R66M, further demonstrating the polymorphic nature of VH1-69 alleles.

### VH1-69 Utilizing mAbs Mediate ADCC Effector Functions but Are Poorly Neutralizing

**Figure 5:**
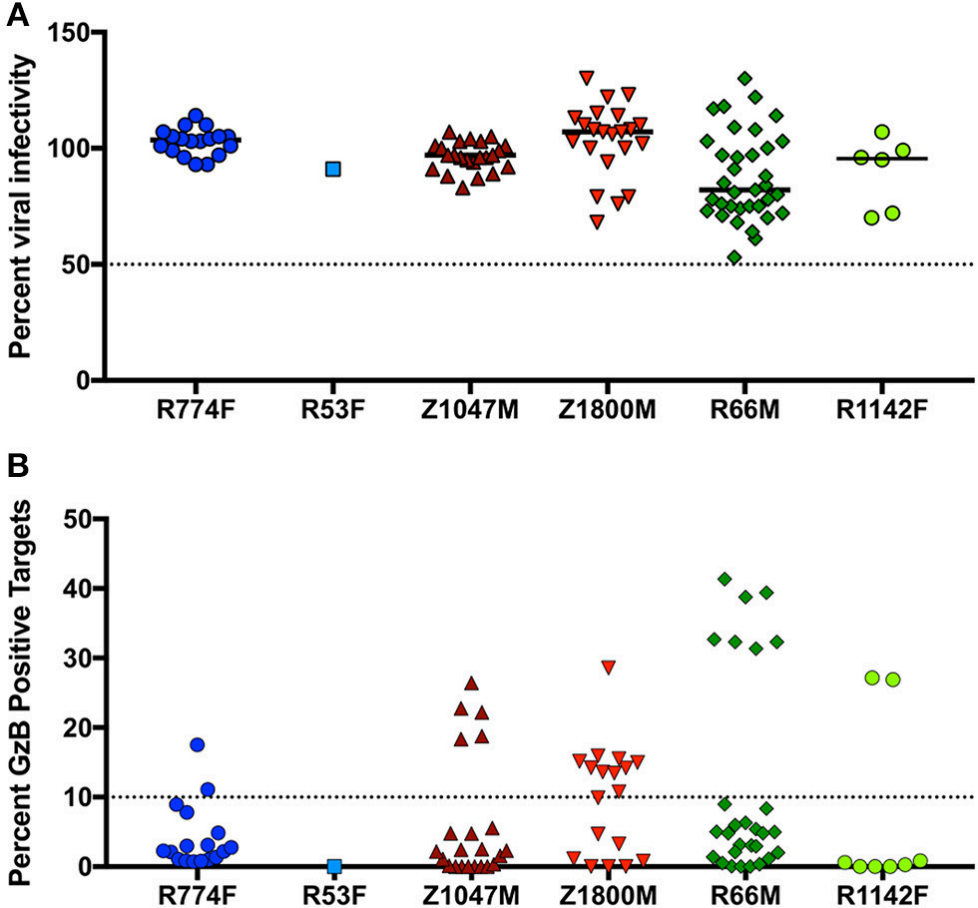
Effector function capabilities of VH1-69 antibodies**. (A)** A concentration of 10 μg/ml of each mAb was evaluated in the TZM-bl neutralization assay against the autologous T/F Env pseudovirus for each individual (labeled below the X-axis). Percent residual infectivity of the T/F Env pseudotyped virus for individual mAbs, relative to virus-only controls, is shown (Y-axis). Symbols represent the mean of triplicate wells, bars represent the median of all mAbs from each individual. **(B)** Each mAb was used in a five-fold dilution series of 5, 1, and 0.2 μg/ml against CEM.NKr.CCR5 cells coated with autologous T/F Env gp120. The peak percentage of cells positive for Granzyme B (GzB) activity over this dilution series, after subtraction of background activity (percentage of GzB positive cells in target + effector reactions), is shown on the Y-axis. Each symbol represents the mean of two separate assays, utilizing effector cells from two independent HIV-negative effector cell donors. The activity of each mAb against target cells coated with the autologous gp120 are labeled for each individual subject below the X-axis. “High” activity was defined as achieving GzB activity >10% (dashed line, Y-axis). Bars indicate the median value for each subject. Raw values are reported in Supplementary Table 4.

In the context of influenza, “innate-like” VH1-69 lineage mAbs can be not only broadly reactive, but broadly neutralizing as well (77). To explore the potential antiviral functions of the dominant anti-gp120 VH1-69 antibody response in early HIV-1 infection, mAbs were produced by cloning VH and VL regions into expression vectors that provided a common IgG1 constant region (53). Thus, all mAbs had identical Fc regions, allowing us to attribute differences solely to the variable domains. Neutralizing activity was present against the autologous T/F Env in five of the six plasma samples contemporaneous to the antibody isolation (Figure 1B). We therefore quantified the neutralization capacity of 105 VH1-69 mAbs against virions pseudotyped with the corresponding autologous T/F Env in the same manner. It should be noted that only one VH1-69 mAb was regenerated from subject R53F, due to the genetics of the B cell response (Figure 2). At 10 μg/ml, none of the mAbs demonstrated potent autologous neutralizing activity, when defined as the ability to reduce infectivity of the T/F Env to below 50% (Figure 5A, Supplementary Table 4). These VH1-69 antibodies are therefore probably not responsible for the observed plasma nAb activity.

Additionally, ADCC activity was also present in the 7.5-month plasma samples in all individuals except R53F (Figure 1B). We therefore evaluated 94 VH1-69 mAbs (overlapping almost completely with the 105 analyzed for nAb activity) for their capacity to mediate ADCC functions in the same manner, using concentrations of 5, 1, and 0.2 μg/ml of each mAb against CEM.NKr.CCR5 cells coated with the autologous T/F Env gp120 (Supplementary Table 4). In contrast to the universally low neutralization capacity exhibited by the VH1-69 mAbs, 27 VH1-69 mAbs mediated high GzB activity, defined as having a peak of GzB positive cells >10% over the course of the dilution series (Figure 5B, Supplementary Table 4), a conservative threshold based on natural variation in “target + effector only” control values between patient-derived gp120s (Supplementary Figure 2). Furthermore, “high GzB” VH1-69 mAbs were present in the same five individuals that exhibited plasma ADCC activity (Figure 1B). The largest proportion of high ADCC-mediating VH1-69 mAbs was observed in Z1800M (56% of tested mAbs), while the highest magnitude was observed in a subset of R66M mAbs (more than 30% peak GzB signaling). As noted above, only one VH1-69 antibody was recovered and tested from subject R53F. Interestingly, R53F was also the only subject that did not have detectable plasma GzB activity at the 7.5-month time point (Figure 1B). Thus, some anti-gp120 VH1-69 mAbs exhibit high ADCC-mediated effector capabilities but poor neutralizing activity in early HIV-1 infection, based on the analysis of memory B cells in these six individuals. These VH1-69 antibodies could contribute to the GzB activity mediated by the contemporaneous plasma.

### High ADCC Activity Is Associated With Shorter CDRH3 Regions and Higher Isoelectric Point in VH1-69 mAbs

Because all of the VH1-69 mAbs studied here have the same Fc region, any differences in the ability to mediate ADCC effector function such as GzB signaling must be attributable to the variable regions. Therefore, all of the VH1-69 mAbs shown in Figure 5B were divided into two groups: those with peak GzB positive cells >10% (after subtraction of background activity), and those below 10%, to search for defining features. When stratified in this manner, there was no statistical difference between the two groups in a number of genetic and functional features, including VH identity to germline, number of tyrosine sulfation sites within CDRH3 (30, 58), CDRH2 GRAVY score, kappa or lambda VL pairing, or binding affinity for the autologous T/F gp120 (Supplementary Figure 3, Supplementary Tables 1, 4). However, there was a significant difference between high and low ADCC VH1-69 antibodies in autologous neutralization capacity, with some of the low GzB antibodies capable of weak autologous neutralization (*p* = 0.0015, Supplementary Figure 3, Supplementary Table 4). It is important to note that, despite this difference, neither group of antibodies was capable of potent autologous neutralization at 10 μg/ml, which is a relatively high concentration (GzB <10% median = 97% infectivity; GzB >10% median = 106% infectivity).

**Figure 6:**
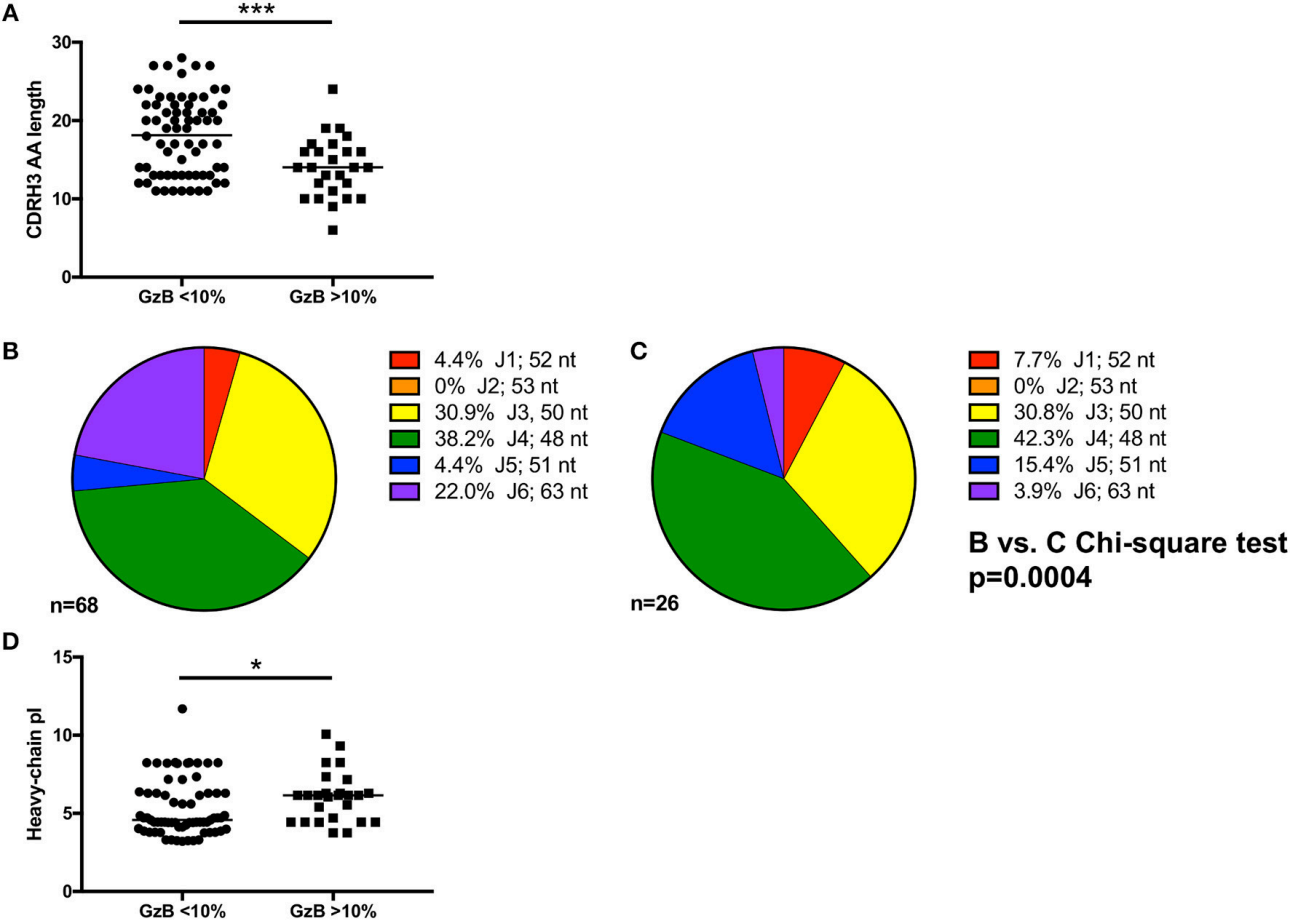
Genetic signatures of VH1-69 mAbs capable of mediating high ADCC activity. **(A)** Amino acid length of mAb complementarity-determining region-3 (CDRH3) (Y-axis) of mAbs with GzB <10% are longer than those from mAbs that mediate high ADCC activity (Mann–Whitney, *p* = 0.0007). **(B)** Distribution of J1 (red), J2 (orange), J3 (yellow), J4 (green), J5 (blue), and J6 (purple) utilization of VH1-69 mAbs that mediated low ADCC activity compared to **(C)** J-region utilization in mAbs that mediated high ADCC activity revealed differential J-region usage between the two groups of mAbs (Chi-square test, *p* = 0.0004). Number of sequences analyzed and percentage of each J-region utilized is indicated. **(D)** A modest difference in isoelectric point (Y-axis) of the heavy chain of the antibodies able to mediate high ADCC vs. low ADCC also emerged (Mann–Whitney, *p* = 0.035). ^*^p ≤ 0.05, ^***^p ≤ 0.001.

In contrast, CDRH3 length did emerge as a defining feature of high Gzb activity, with antibodies capable of mediating high ADCC activity having statistically shorter CDRH3 amino acid length (*p* = 0.0007, Figure 6A, Supplementary Tables 1, 2). However, the median CDRH3 length for these high GzB antibodies was not unusually short compared to the median length determined for rearranged human B cells (median CDRH3 length for GzB >10% = 14 AA vs. reported median value = 15 AA) (70). This observation suggests that long CDRH3 regions may be less conducive to antibody-Fc interactions as measured in this setting.

To further investigate the association of CDRH3 length and high ADCC function, we focused on the impact of the J region, which is selected during VDJ rearrangement and contributes to the length of CDRH3. While there are six J_H_ regions in the human repertoire, it is striking that bnAbs commonly use the longest J region, J6, including b12, 3BNC117, 8ANC131, PGT145, PG9, PGT121, PGT151, and 2F5 (16). We therefore examined J region usage in the VH1-69 mAbs to gain a better understanding of how CDRH3 length was generated. Using the VH1-69 mAbs grouped by low or high GzB activity as shown in Figure 6A, similar J1, J3, and J4 usage was found between the two groups (Figure 6B low and Figure 6C high, Supplementary Tables 2, 4). Conversely, J6 was more frequently utilized by low GzB antibodies (22.0%) compared to the high GzB group (3.9%), the latter of which had higher J5 usage. None of the VH1-69 mAbs contained J2. The overall distribution of J chains was significantly different between the two groups of VH1-69 antibodies (Chi-square, *p* = 0.0004, J2 was excluded due to the zero frequency). Thus, the infrequent presence of J6 could contribute to shorter CDRH3 regions in this antibody population. There was also a significant difference in the calculated isoelectric point (pI) for the heavy chain of the antibodies able to mediate high GzB activity vs. those that did not (*p* = 0.035, Figure 6D, Supplementary Table 2). The pI is the pH at which the antibody has no net charge, and can be estimated based on the amino acid sequence. pI can influence such parameters as tissue distribution and clearance *in vivo*. Interestingly, mAbs that mediated higher GzB activity exhibited a higher, closer to neutral, median isoelectric point (4.58 vs. 6.15). Thus, at a physiological pH, the high GzB mAbs would be less negatively charged than the low GzB mAbs, perhaps influencing their recognition of certain epitopes. Overall, this analysis revealed that VH1-69 antibodies that mediate higher GzB activity against target cells coated with the autologous T/F Env gp120 protein have unique features that include relatively shorter CDRH3 regions, associated with J chain preference, and a closer to neutral isoelectric point than those with low GzB activity.

### mAb Epitope Does Not Define High Autologous ADCC Activity

**Figure 7:**
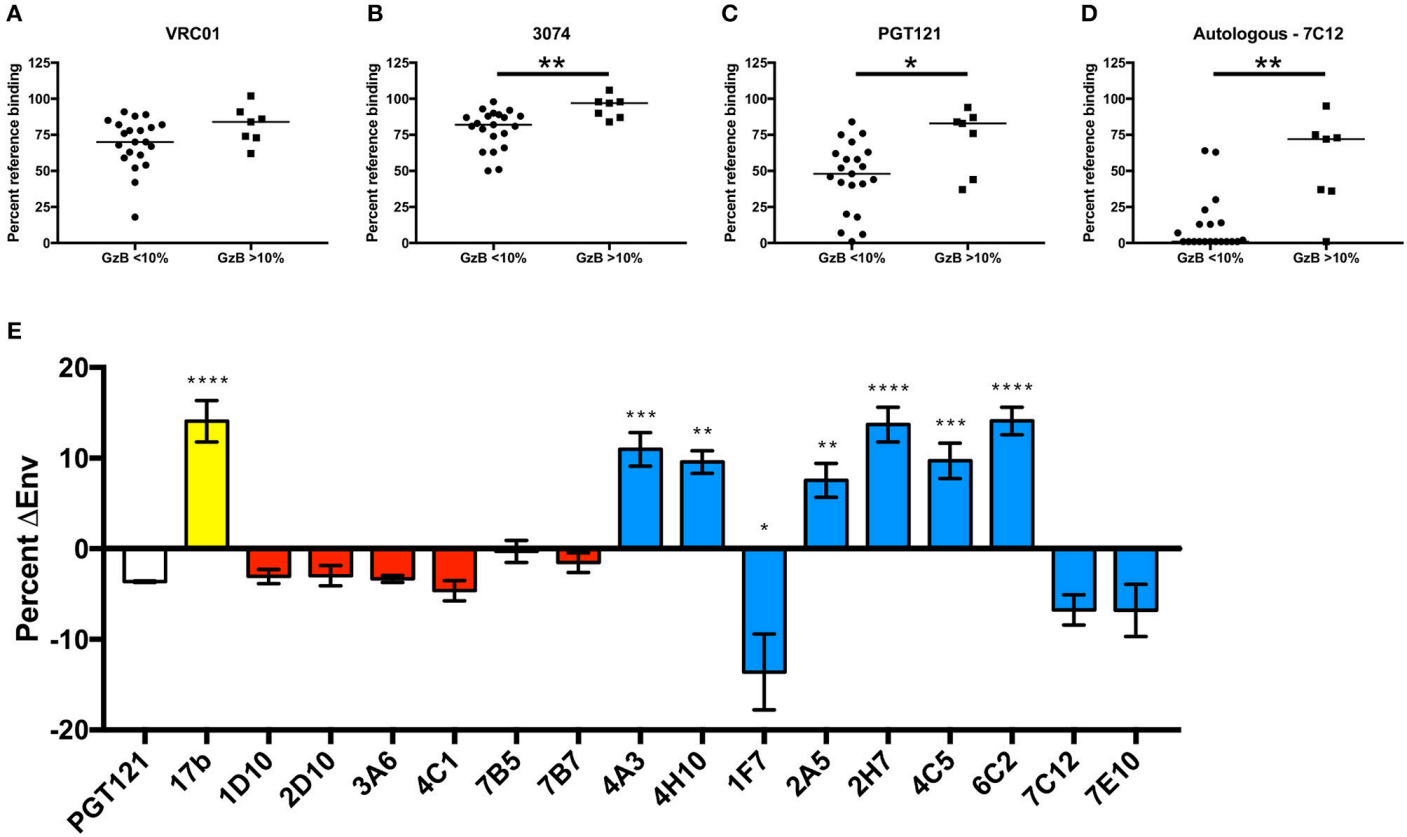
Definition of putative epitopes of R66M VH1-69 mAbs through competition. In order to gain insight into the putative T/F Env gp120 epitopes targeted by R66M VH1-69 mAbs, the ability of low ADCC (GzB <10%) and high ADCC (GzB>10%) mAbs to reduce binding of reference mAbs **(A)** VRC01 (CD4 binding site), **(B)** 3074 (V3), **(C)** PGT121 (N332-glycan supersite, V3 base), and **(D)** a “median” autologous R66M mAb 7C12 to the R66M T/F gp120 protein was assessed via epitope binning on the Octet RED96. Percent residual binding of the reference antibody (top label) in the presence of each tested R66M mAb is reported, bar indicates median value. Statistically significant differences between low and high ADCC mAbs was observed for competition with 3074 (Mann–Whitney, *p* = 0.005), PGT121 (*p* = 0.02) and with the autologous mAb, 7C12 (Mann–Whitney, *p* = 0.002). **(E)** CD4bs and CD4i epitopes were identified via quantification of R66M Env-positive transfected 293T cells in the absence and presence of sCD4. Y-axis represents the increase (positive values) or decrease (negative values) of Env-positive cells in the presence of sCD4 relative to no sCD4. Binding of bnAb PGT121 (white bar) to R66M Env is not substantially affected by sCD4, thus was used as a reference. Control CD4i mAb 17b binding (yellow bar) significantly increased when transfected cells were pre-incubated with sCD4 (Ordinary one-way ANOVA, *p* = 0.0001). R66M high ADCC mAbs (red bars) were not significantly affected by sCD4, however several low ADCC mAbs (blue bars) demonstrated likely CD4i epitopes (4A3, *p* = 0.0004; 4H10, *p* = 0.0014; 2A5, *p* = 0.0047; 2H7, *p* = 0.0001; 4C5, *p* = 0.0005; 6C2, *p* = 0.0001) or CD4bs epitope (1F7, *p* = 0.027). ^*^p ≤ 0.05, ^**^p ≤ 0.01, ^***^p ≤ 0.001, ^****^p ≤ 0.0001.

To investigate the epitopes that were targeted on gp120 by high ADCC-mediating mAbs, a series of binding competition experiments were carried out with VH1-69 mAbs from the individual with the highest ADCC signaling, R66M. We utilized bnAbs with known specificities against the CD4bs, V3, and the mannose patch/base of V3 to gain information about the R66M mAb epitopes. CD4bs mAbs are thought to be unable to mediate ADCC in the gp120 coating assay because the protein is bound to cell-surface CD4. Therefore, to test this hypothesis, 28 R66M VH1-69 mAbs were assessed for their ability to compete with the CD4bs bnAb VRC01 for binding to the R66M T/F gp120 protein using biolayer interferometry. Overall, the majority of VH1-69 mAbs from R66M did not compete with VRC01. Only 2 of 28 mAbs reduced VRC01 binding to <50% (Figure 7A, Supplementary Table 4). As expected, these two antibodies belonged to the low GzB signaling group; however, there was no statistical difference between the two groups in their ability to compete with VRC01 binding (Figure 7A).

We next tested competition against a V3-directed bnAb, 3,074, that recognizes a linear epitope conserved among HIV-1 clades A, B, and C (78). In general, the VH1-69 mAbs recovered from R66M competed only weakly 3,074 (Figure 7B, Supplementary Table 4). However, the level of V3-directed competition was greater in the low GzB group (Figure 7B, Mann–Whitney, *p* = 0.005). Thus, these findings contradict the idea that CD4 binding in the gp120-coating assay preferentially measures V3-mediated GzB activity (Figure 7B). Furthermore, none of the six high GzB mAbs from R66M appeared to target V3.

The R66M mAbs were next competed against bnAb PGT121, which recognizes an epitope formed by V3-proximal glycans and the V3 base. A relatively large proportion of R66M VH1-69 mAbs competed to varying degrees with PGT121 for gp120 binding. In terms of GzB activity, 13 mAbs were capable of reducing PGT121 binding to <50%, including two from the high GzB group and 11 from the low GzB group. A significant difference in PGT121 competition was observed between the two groups (Figure 7C, *p* = 0.027). Thus, antibodies with epitopes overlapping that of PGT121 did not commonly mediate high GzB activity. However, two R66M VH1-69 antibodies that competed with PGT121 did mediate high Fc-mediated GzB activity. This finding is significant in that it demonstrates that mAbs arising in the same individual can recognize a similar epitope on gp120 yet some exhibit high ADCC activity while others do not.

To explore this concept further, VH1-69 mAbs from R66M were competed against an autologous mAb 7C12, which was chosen because its CDRH3 length was the median for R66M (20 AA), it exhibited high affinity binding to the R66M T/F gp120, and this mAb lacked ADCC activity. Many of the VH1-69 R66M mAbs competed with 7C12 (75% of all those tested) indicating that they likely recognize overlapping epitopes. When stratified by GzB activity, the VH1-69 mAbs in the low GzB group competed with 7C12 to a greater degree than the high GzB group (Figure 7D, *p* = 0.006). However, three mAbs in the high GzB group also strongly reduced 7C12 binding (Figure 7D, Supplementary Table 4: mAbs 1D10, 6C12, and 7B7). These findings provide additional evidence that the angle of binding and other factors extrinsic to the epitope are important for mediating Fc functions. They further suggest that a novel, immunodominant epitope exists within the R66M gp120 protein that is highly susceptible to autologous ADCC activity.

Additionally, an important observation from these studies is that the gp120 coating assay detected high Fc-mediated GzB activity by antibodies with epitopes that are not expected to require induction by CD4, such as those antibodies competing with PGT121 (Figure 7C). This was somewhat unexpected due to the nature of the assay (gp120 pre-bound to surface CD4), and the preponderance of previously described CD4i mAbs that utilize VH1-69, including 17b, c12/SB1/X5, 23e, 47e/412d/E51, and M16 (30). To investigate more directly whether the six R66M VH1-69 mAbs capable of mediating high GzB signaling within this assay are enriched for CD4i epitopes, we first assessed the ability of 16 R66M mAbs to recognize endogenously produced, processed, membrane-bound autologous T/F Env presented on the surface of transfected 293T cells. This panel of mAbs included six that mediated high GzB activity and nine that did not. All of the R66M mAbs tested were capable of recognizing membrane bound, autologous R66M T/F Env except for mAb 6C12, which did mediate ADCC and clearly bound to soluble gp120 in OctetRED and ELISA assays (ELISA data not shown) (Supplementary Figure 4).

We next determined whether binding of the remaining 15 mAbs to surface expressed Env was influenced by addition of soluble human CD4 (sCD4). Flow cytometry was performed with R66M mAbs from the high (*n* = 6) and low (*n* = 9) GzB groups to determine the percent of Env-positive 293T cells in the absence and presence of sCD4. Differences in binding, whether an increase (suggestive of a CD4i epitope) or decrease (suggestive of a CD4bs epitope) were compared to PGT121 binding, which is not sensitive to the addition of sCD4. Binding of mAb 17b, which recognizes a CD4i epitope, was used for comparison (Figure 7E). Interestingly, the CD4i antibody 17b bound to surface expressed R66M Env in the absence of sCD4, perhaps suggesting this Env trimer is natively in a more “open” conformation. However, 17b binding was significantly increased more than 10% upon pre-incubation of the Env-expressing cells with sCD4 (Figure 7E). Remarkably, binding of the six R66M mAbs capable of mediating high ADCC activity (red) to surface-expressed Env was not enhanced by sCD4, while several low ADCC mAbs (blue) did display altered binding profiles in the presence of sCD4 (Figure 7E). This latter group included mAb 1F7, which had reduced binding in the presence of sCD4 and also competed with VRC01, although it did not mediate potent neutralization. There were also several mAbs that displayed a significant increase in binding in the presence of sCD4, similar to 17b, yet none of these mAbs mediated high ADCC activity in the gp120 coating assay.

## Discussion

VH1-69 utilizing antibodies have gained notoriety as first-responders in a number of viral infections. An over representation of VH1-69 antibodies has been described in influenza infection, hepatitis C associated cancers, respiratory syncytial virus infection, and HIV-1 infection (70, 77, 79–82). A widely accepted explanation for the ability of VH1-69 antibodies to readily interact with diverse viral proteins is the inherent hydrophobic CDRH2 region that is unique to VH1-69 alleles (30, 75). Here, we also observed an over representation of VH1-69 germline-utilizing antibodies that recognize autologous T/F gp120 proteins, in five of six individuals infected with diverse HIV-1 variants prevalent in Sub-Saharan Africa (Subtype A, C, and A/C recombinants). A unique feature of our study is that it was carried out at ~7-months post-infection and demonstrates that the VH1-69 response occurs early. The broad predominance of anti-gp120 VH1-69 antibodies in the subjects studied here, infected with diverse HIV-1 variants, prompted us to delve deeper into the features and antiviral functions of individual VH1-69 mAbs. Within ~100 VH1-69 mAbs from the six HIV-1 infected individuals that were tested for *in vitro* Fc-mediated GzB activity, nearly one-third were able to mediate high ADCC activity against cells coated with autologous T/F gp120. In contrast, none of the VH1-69 antibodies were capable of potent mAb neutralization capacity. Importantly, despite the hypothesis that VH1-69-utilizing mAbs are “innate like,” the absence of substantial ADCC activity in the plasma near the time of infection illustrates affinity maturation is required to acquire effector functions.

The VH1-69 mAbs in our study also exhibited CDRH2 regions that were significantly more hydrophobic than the non-VH1-69 anti-gp120 sequences from the same individuals. This observation is consistent with the concept that VH1-69 alleles have evolved in humans to provide a “first-line response” to viral infections (83). The VH1-69 locus itself is genetically diverse, with at least 14 alleles described thus far, and copy number variation from 2 to 4 copies per individual (2, 76, 84–86), perhaps hinting that it might be under selective pressure to continue to diversify within the human population. Further supporting this is the observation that genetic variation within this locus can influence the anti-viral response. In the context of human influenza infection, antibodies that contain phenylalanine at amino acid position 54 are associated with neutralization, while those that contain leucine at this position are not only infrequently induced, but also lack neutralization capacity in comparison to the 54F variants (2, 76). If this division in function remained prominent for other viral infections, such as HIV-1, it could have substantial implications for vaccine design, as these alleles are differentially globally distributed (2). While VH1-69 allelic variation has not been exhaustively defined, especially in African populations relevant to the individuals studied here, we were able to quantify the number of anti-gp120 VH1-69 sequences that contained F, L, or another amino acid at position 54. As previously reported, the 54F-containing sequences were the most frequently utilized, and generally composed the majority of VH1-69 antibodies even when the 54L-containing sequences were also present in the same individual. The one exception to this observation was R66M, where 54L-containing alleles were the dominant VH1-69 genotype. In contrast to influenza infection, where the 54L genotype results in fewer VH1-69 anti-viral antibodies, in R66M, anti-gp120 VH1-69 utilizing antibodies containing both 54F and 54L made up 51% of the isolated sequences (76). This suggests that the dearth of 54L VH1-69 antibodies observed in individuals during influenza infection does not necessarily translate to a similar bias in HIV-1 infection. Also in contrast to influenza infection, where the 54F genotype is associated with neutralization, the ability to mediate neutralization or ADCC effector functions against HIV-1 was not stratified by VH1-69 genotype (76). Both 54F and 54L were found in the high and low ADCC-mediating groups. This could be a reason for cautious optimism, in the context of vaccines specifically designed to elicit polyfunctional antibody responses, that include ADCC effector functions in humans (87). However, VH1-69 allelic diversity is only one component of host genetics that influences ADCC. Other factors likely influence ADCC *in vivo*, such as FcγRIIIa allelic variation (88, 89). For example, polymorphisms present at position 158 in FcγRIIIa, such as valine (V) or phenylalanine (F), can differ in affinity for antibody Fc. This could alter the potency and efficacy of ADCC *in vivo*, even if appropriate ADCC-mediating antibodies are present. The assay utilized here to measure ADCC activity included effector cells from HIV-negative donors, who were screened to eliminate low affinity (F/F homozygous) donors. Though these and other differences in host genetics could alter the performance of these antibodies *in vivo*, we have identified and described a number of antibodies, with distinct genetic features, present in five of six individuals, that have the potential to mediate autologous ADCC effector function against HIV-1 T/F gp120. These antibodies also bind to endogenously expressed Env on the cell surface, and are not dependent on the presence of CD4 to bind to their epitope or mediate Fc-mediated GzB signaling. It will be of interest to further define the novel epitopes that are susceptible to ADCC, whether they are conserved across HIV-1 variants, and what features of Env are important to elicit them.

Interestingly, a number of genetic and biochemical features did not distinguish VH1-69 antibodies capable of mediating high ADCC activity from those that did not. Importantly, and supporting the idea that VH1-69 interactions with viral glycoproteins are innate-like, there was no difference in somatic hypermutation (i.e., percent identity to germline) and likewise, no difference in binding affinity for the autologous T/F gp120 protein between VH1-69 ADCC-mediating and non-mediating antibodies. Thus, it was not necessarily routine selection and affinity maturation that lead to mAbs capable of ADCC, nor generic “ELISA-like” binding of antibody to gp120 that resulted in ADCC effector functions observed here. All of the VH1-69 antibodies bound to soluble gp120, but only a subset could mediate ADCC using the exact same protein coated onto the target cell surface. A possible explanation is that some mAbs bind to gp120 at an angle that facilitates a sufficient number of antibody-Fc interactions with effector cells needed to trigger granzyme B release, i.e., an “angle of attack” conducive to mediating ADCC functions, while other antibodies bind in a manner that was not favorable for Fc receptor engagement. Though structural studies will be necessary to fully appreciate the importance of specific epitopes and angle-of-binding to those epitopes, we describe clear instances where antibodies that share similar epitopes display divergent ADCC activity. Similar findings were reported in previous analyses of anti-HIV ADCC activity, where the importance of not only the recognized epitope, but also the angle of antibody binding, was emphasized (90). Certainly, this is an important observation in the context of HIV-1, but it is also an important concept to consider when generating any therapeutic mAb with a desired ADCC mechanism of action.

A genetic feature that was strongly associated with high ADCC activity in this study was shorter CDRH3 length. In stark contrast with bnAbs, which sometimes include exceptionally long CDRH3 regions, VH1-69 mAbs capable of mediating ADCC had, on average, shorter CDRH3 regions than non-mediating mAbs (16). However, these “short” CDRH3s averaged 14 AA in length, near the expected average lengths of CDRH3 in “control” human antibodies [reported value 15 AA 70]. This “average” CDRH3 length appeared to be related to a lower frequency (but not absence) of J6 usage in the high ADCC mAb group, which can contribute to longer CDRH3 length [63 nt addition, 91] and is frequently found in bnAbs with long CDRH3 (16). The infrequent use of J6 was compensated for mainly by a three-fold increase in J5 usage, which is of average size (51 nt addition). An additional genetic metric that was modestly distinctive of high ADCC mAbs was a higher isoelectric point of the heavy chain that was closer to neutral. The isoelectric point of mAbs has been strongly associated with their *in vivo* clearance and half-life (92). Antibodies mutated within the VH to have lower isoelectric points exhibited improved half-lives over the original unmutated mAbs, without modifying the constant region or light chain (92). Considering the lack of protection observed in non-human primate studies that utilized non-neutralizing antibodies (93, 94), it would be tempting to hypothesize that targeted reduction of isoelectric point of established non-neutralizing, ADCC-mediating antibodies could potentially improve the outcome of these trials. Thus, the data presented here begin to generate a picture of antibodies that can mediate ADCC-mediated functions against the autologous Env, providing tangible features that distinguish them from non-ADCC antibodies. Furthermore, these findings highlight three important points. First, the R66M mAbs that mediated high ADCC activity were generally able to recognize membrane-bound Env in the absence of CD4, in addition to their capacity to bind to soluble gp120. Secondly, the high ADCC-mediating mAbs were not directed toward CD4i epitopes, thus this experimental approach was not biased to detect Fc-mediated activity against epitopes exposed by gp120 binding to CD4. Finally, the recognition of CD4i epitopes was not the primary determinant of Fc-mediated GzB activity for R66M mAbs in the gp120 coating assay. Together, these findings suggest that there are many more facets of Fc-mediated antibody activity than are currently appreciated, and that the use of varied *in vitro* assays and multiple approaches can enrich this body of knowledge. Furthermore, the use of novel antibodies and autologous antibody/protein combinations may be key to furthering these discoveries of ADCC epitopes and antibody binding modes.

The data presented here can be considered in the context of unanswered questions. For example, it remains to be seen what impact these ADCC antibodies have on the viral population and disease progression. Plasma neutralization capacity can clearly influence the evolution of an individual's viral quasispecies, while HIV-1 Env's capacity to tolerate mutations and maintain viral fitness makes the influence that nAb and has on disease progression and pathogenesis is less clear (95–97). There is also the question as to whether these mAbs with ADCC function, present pre-exposure, could reduce transmission rates or improve disease progression post-infection using an autologous challenge virus. Alternatively, there is a possibility, especially within the context of the ADCC assay utilized here, that ADCC mAbs present as pre-existing immunity could have a detrimental effect on disease progression and CD4+ T-cell counts, as soluble gp120 could adhere to the surface of uninfected CD4+ T-cells and target them for cell death (though it should be noted that in the referenced study, uninfected “bystander cells” could not be differentiated from cells bound to virions, in the process of becoming infected) (98). It will also be interesting to investigate whether any of these early autologous ADCC-mediating mAbs, with conservative identities to germline and average affinities for autologous T/F gp120, display ADCC breadth, or whether they display strain specificity, which is frequently observed with early autologous neutralizing antibodies. The ability to recognize and exert multiple antiviral functions against a broad range of genetically and geographically distinct HIV-1 variants is almost certainly required for protection against infection. Although only autologous nAb and ADCC activity were assessed in the current study, we have identified common genetic features of early VH1-69 anti-gp120 antibodies induced in distinct host genetic backgrounds against diverse HIV-1 variants that are associated with higher GzB activity. Future studies will be required to determine whether these antibodies are cross-reactive and if they can also mediate ADCC functions in other assays. The dominance of VH1-69 in these HIV-1 infected individuals suggests an immunologic convergence that could be harnessed to elicit antibodies capable of mediating ADCC activity against gp120, suggesting that a better understanding of this process could inform the design of vaccine immunogens. Understanding the complexities of how ADCC mediating antibodies arise in infected and vaccinated individuals, and carry out effector functions, will likely require the use of varied *in vitro* assays and multiple approaches. The discovery of new antibodies with novel epitopes and binding modes will facilitate this process, expanding our knowledge beyond commonly used prototypical antibodies and viral strains.

## Author Contributions

CD and SS: conceptualization, methodology, and writing—original draft; CD, SS, and SB: formal analysis; CD: funding acquisition, project administration, and supervision; SS and SB: investigation; WK, SL, EK, MP, and SA: resources; CD, SS, WK, SL, EK, MP, and SA: writing—review & editing.

### Conflict of Interest Statement

The authors declare that the research was conducted in the absence of any commercial or financial relationships that could be construed as a potential conflict of interest.

## Supplementary Material

Supplementary Table 1 Genetic characterization of paired heavy- and light-chain sequences isolated from autologous T/F gp120 reactive single memory B cells sorted from PBMC collected at 7.5 months after infection (Time Point 2). Sequences from paired heavy- and light-chains from autologous T/F Env gp120-reactive single B cells were analyzed via IMGT vquest (http://imgt.org/IMGT_vquest/vquest) to determine (i) Variable heavy (VH) germline and allele, (ii) VH identity to germline, (iii) CDRH2 sequence determination, (iv) CDRH3 length, (v) CDRH3 sequence determination, (vi) Variable light (VL) germline and allele. CDRH2 GRAVY scores were calculated via (http://www.gravy-calculator.de).

Supplementary Table 2 Genetic features of VH1-69 utilizing antibody sequences. VH1-69 sequences were analyzed in further detail, due to their overrepresentation in isolated anti-gp120 memory B cells. Variable Joining region (VJ) alleles and heavy-chain isoelectric points were determined via IMGT vquest (http://imgt.org/IMGT_vquest/vquest). Amino acid 54 within CDRH2 is highlighted in bold. CDRH2 GRAVY scores were calculated via (http://www.gravy-calculator.de). The number of tyrosine sulfination sites contained within CDRH3 was quantified via the Sulfinator online tool (http://web.expasy.org/sulfinator/) (58).

Supplementary Table 3 Quantification of VH1-69 mAb binding affinity for autologous T/F gp120. The affinity of VH1-69 utilizing mAbs for binding to the autologous T/F Env gp120 protein was determined on an Octet RED96. The molarity of gp120 utilized for measurement, calculated KD and KD error, kon and kon error, kdis and kdis error, X^2^, and R^2^ values are reported. Average KD, kon, and kdis values are reported for each set of measurements. Median X^2^ (and range), R^2^ (and range), KD, kon, and kdis are reported for each individual.

Supplementary Table 4 Neutralizing and non-neutralizing Fc-mediated effector function of VH1-69 utilizing antibodies. The ability of virions pseudotyped with autologous T/F Env to infect TZM bl indicator cells in the presence of 10 μg/ml of each mAb was assayed in triplicate, and repeated independently as quantity allowed. Non-neutralizing Fc-mediated effector function was quantified via GranToxiLux assay, in a mAb dilution series of 5, 1, and 0.2 μg/ml against CEM.NKr.CCR5 cells coated with autologous T/F Env gp120. Values reported are the mean peak percentage of cells positive for Granzyme B (GzB) activity over this dilution series, after subtraction of background activity, utilizing effector cells from two independent HIV-negative effector cell donors.

Supplementary Figure 1 Representative Granzyme B control and experimental flow-plots. CEM.NKr.CCR5 cells were pulsed with gp120 proteins (R66M gp120 illustrated here). After gating for size (targets) and NFL4 negative (live) cells, determination of Granzyme B activity was calculated by drawing gates based on target cells incubated alone (Targets Alone). The percentage calculated within “Targets + Effectors” was inferred to be background GzB activity, and subtracted from all other positive percentages to calculate final positive percentage. Negative and Positive controls were included in all assays. In groups of tested patient derived mAbs, ADCC high (R66M 1D10) and low (R66M 1F7) were observed.

Supplementary Figure 2 Impact of T/F Env gp120 proteins and effector cell donor variation on ADCC and autologous plasma control assays. The percentage of GzB positive cells between individuals was compared in reactions containing **(A)** only T/F Env gp120 coated CEM.NKr.CCR5 target cells and donor effector cells (background GzB activity), **(B)** negative control 50 μg/ml commercial polyclonal IgG (minus background), and **(C)** positive control 50 μg/ml HIV-Ig (minus background). There were no significant differences in these parameters between the six T/F Env gp120 proteins (Kruskal–Wallis, ns), although there was ~2- to 3-fold variation in HIV-Ig responses as would be expected. Bars illustrate standard error of the mean. **(D)** There was no significant difference between GzB percentages between results obtained using Donor 190 and 457 effector cells (Mann–Whitney, ns), and **(E)** the GzB results were highly correlated between the two donors (Spearman, *p* < 0.0001, *r* = 0.82). **(F)** Negative control data for Figure 1. nAb and ADCC activity in plasma collected at chronologic Time Points 1, 2, and 3; (i) nAb against a negative control VSV-g Env pseudovirus (closed symbols, solid lines), and (ii) GzB mediated by normal human serum against the T/F Env gp120 (open symbols, dashed lines). Symbols and error bars represent the mean and standard error of mean, respectively, between at least two replicate experiments for neutralization, and the mean and standard error of mean of independent experiments utilizing cells from two different donors for the GzB assay.

Supplementary Figure 3 Genetic and functional comparison of VH1-69 low ADCC mAbs vs. high ADCC mAbs. VH1-69 mAbs were stratified into two groups, based on low (peak GzB percentage <10%) or high (peak GzB percentage >10%) ADCC activity. No significant differences emerged between these two groups in a number of metrics: **(A)** heavy-chain percent nucleotide identity to germline (Mann–Whitney, *p* = 0.74); **(B)** the number of tyrosine sulfation sites found within the heavy-chain complementarity-determining region-3 (CDRH3) (Mann–Whitney, *p* = 0.18) (30); **(C)** kappa or lambda light-chain pairing (Fisher's exact test, *p* = 0.38); **(D)** heavy-chain complementarity-determining region-2 (CDRH2) grand average hydropathy (GRAVY) scores (Mann–Whitney, *p* = 0.89); **(E)** binding affinity for autologous T/F gp120 (Mann–Whitney, *p* = 0.76). **(F)** There was a significant difference between the two groups and their ability to neutralize virions pseudotyped with the autologous T/F Env (Mann–Whitney, *p* = 0.0015). ^**^*p* ≤ 0.01.

Supplementary Figure 4 Antibody recognition of R66M T/F Env transfected cells in the absence and presence of sCD4. 293T cells were transfected with R66M T/F Envelope expression plasmid. 48-h post-transfection, cells were stained with control and R66M VH1-69 mAbs (indicated above plots), either **(A)** without or **(B)** with pre-incubation with sCD4. X-axis indicates PE intensity, Y-axis indicates side scatter area (SSC-A). “Env-positive” cells are found in the lower right quadrant (Q3), percentage of total events per quadrant are indicated. **(C)** Percent Env-positive cells, minus IgG background, for each mAb in the absence (white) and presence of sCD4 (gray). Columns indicate mean of a minimum of three independent experiments, bars represent SEM.
