# Supplementary figures and images for: VH1-69 Utilizing Antibodies Are Capable of Mediating Non-neutralizing Fc-Mediated Effector Functions Against the Transmitted/Founder gp120
Source: Front Immunol. 2019 Jan 15;9:3163. doi: 10.3389/fimmu.2018.03163 (PMC6341001; doi:10.3389/fimmu.2018.03163)

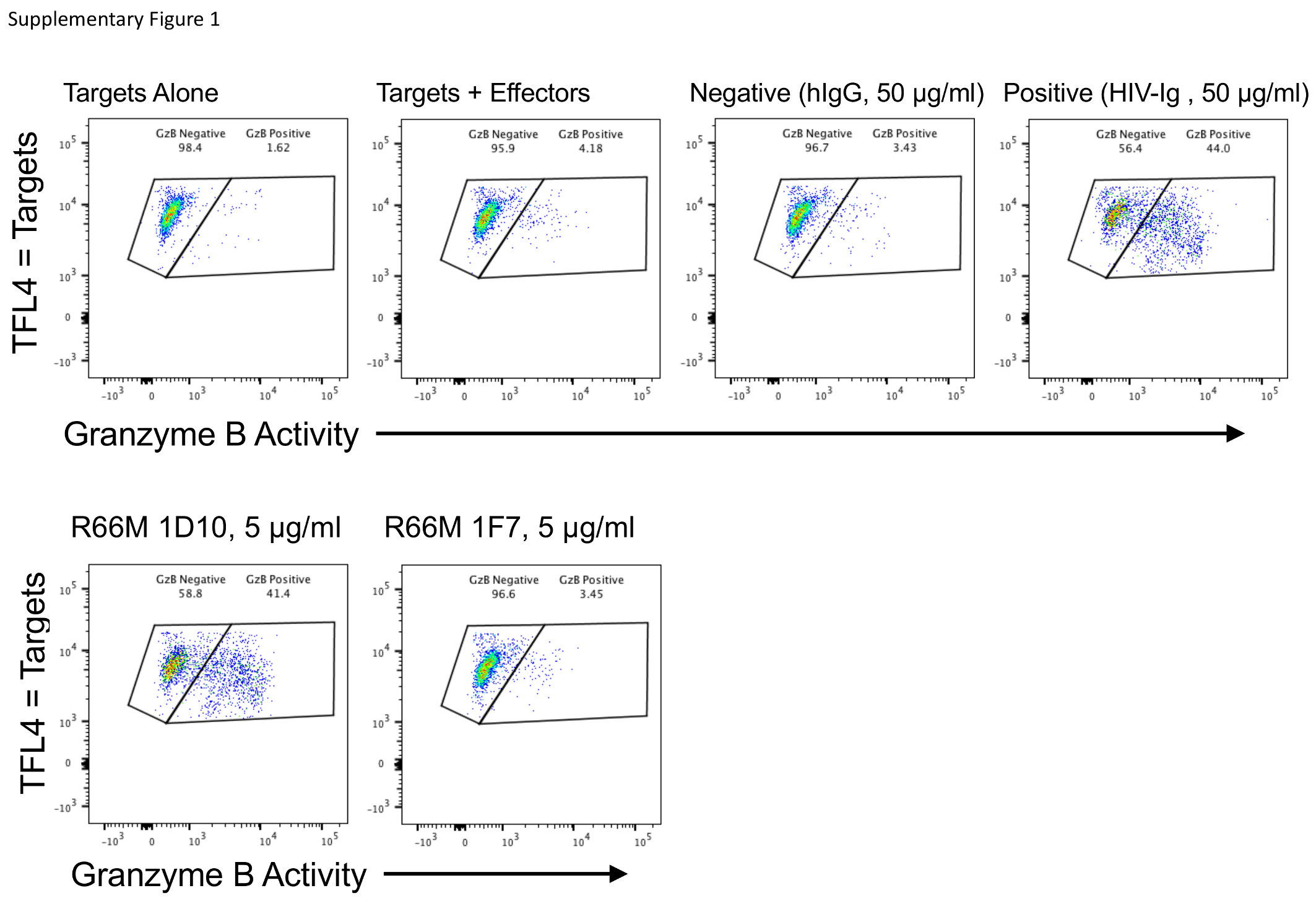

Supplement: Supplementary Figure 1 — Representative Granzyme B control and experimental flow-plots. CEM.NKr.CCR5 cells were pulsed with gp120 proteins (R66M gp120 illustrated here). After gating for size (targets) and NFL4 negative (live) cells, determination of Granzyme B activity was calculated by drawing gates based on target cells incubated alone (Targets Alone). The percentage calculated within “Targets + Effectors” was inferred to be background GzB activity, and subtracted from all other positive percentages to calculate final positive percentage. Negative and Positive controls were included in all assays. In groups of tested patient derived mAbs, ADCC high (R66M 1D10) and low (R66M 1F7) were observed. [file Image_1.TIF]

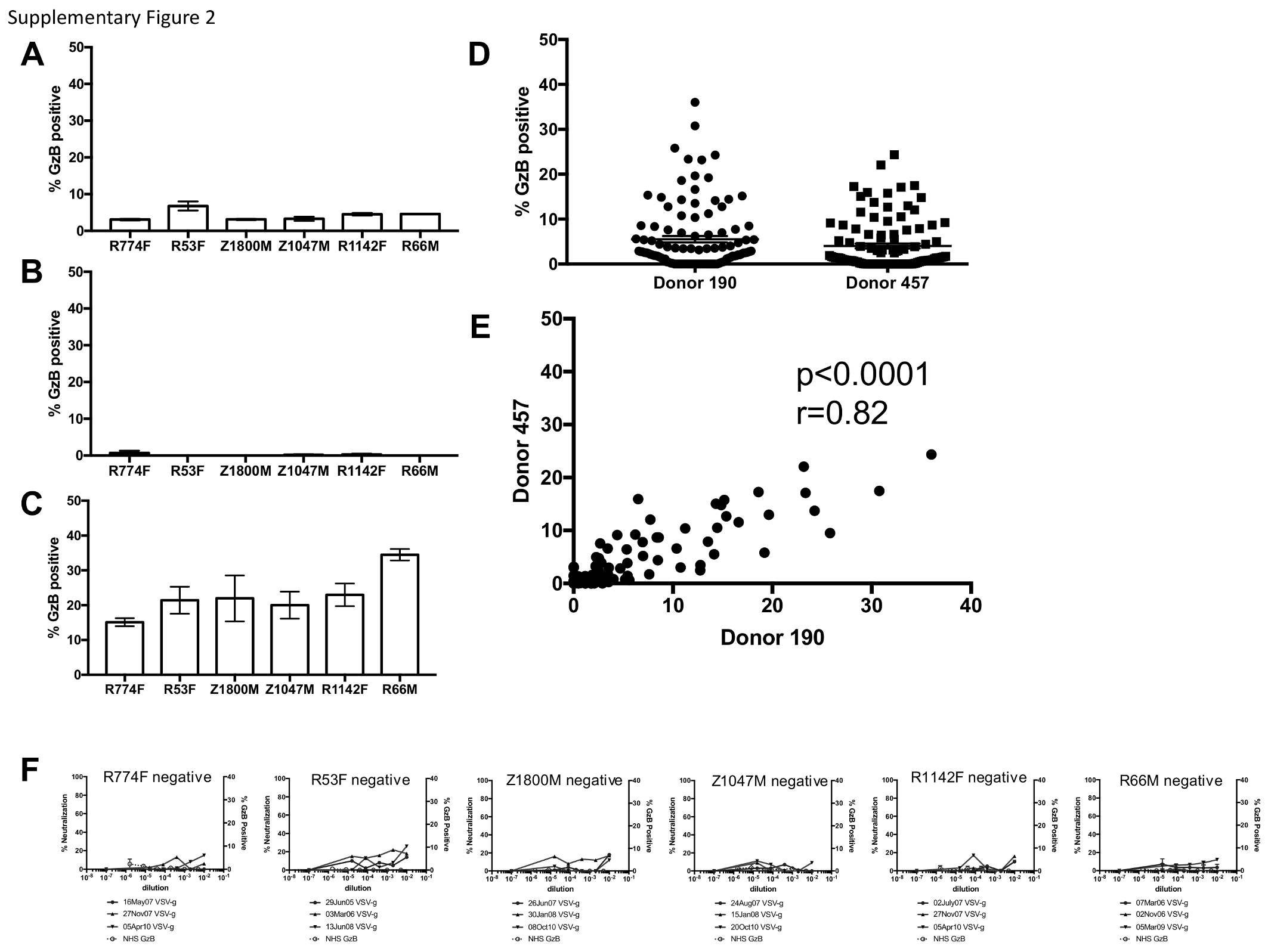

Supplement: Supplementary Figure 2 — Impact of T/F Env gp120 proteins and effector cell donor variation on ADCC and autologous plasma control assays. The percentage of GzB positive cells between individuals was compared in reactions containing (A) only T/F Env gp120 coated CEM.NKr.CCR5 target cells and donor effector cells (background GzB activity), (B) negative control 50 μg/ml commercial polyclonal IgG (minus background), and (C) positive control 50 μg/ml HIV-Ig (minus background). There were no significant differences in these parameters between the six T/F Env gp120 proteins (Kruskal–Wallis, ns), although there was ~2- to 3-fold variation in HIV-Ig responses as would be expected. Bars illustrate standard error of the mean. (D) There was no significant difference between GzB percentages between results obtained using Donor 190 and 457 effector cells (Mann–Whitney, ns), and (E) the GzB results were highly correlated between the two donors (Spearman, p < 0.0001, r = 0.82). (F) Negative control data for Figure 1. nAb and ADCC activity in plasma collected at chronologic Time Points 1, 2, and 3; (i) nAb against a negative control VSV-g Env pseudovirus (closed symbols, solid lines), and (ii) GzB mediated by normal human serum against the T/F Env gp120 (open symbols, dashed lines). Symbols and error bars represent the mean and standard error of mean, respectively, between at least two replicate experiments for neutralization, and the mean and standard error of mean of independent experiments utilizing cells from two different donors for the GzB assay. [file Image_2.TIF]

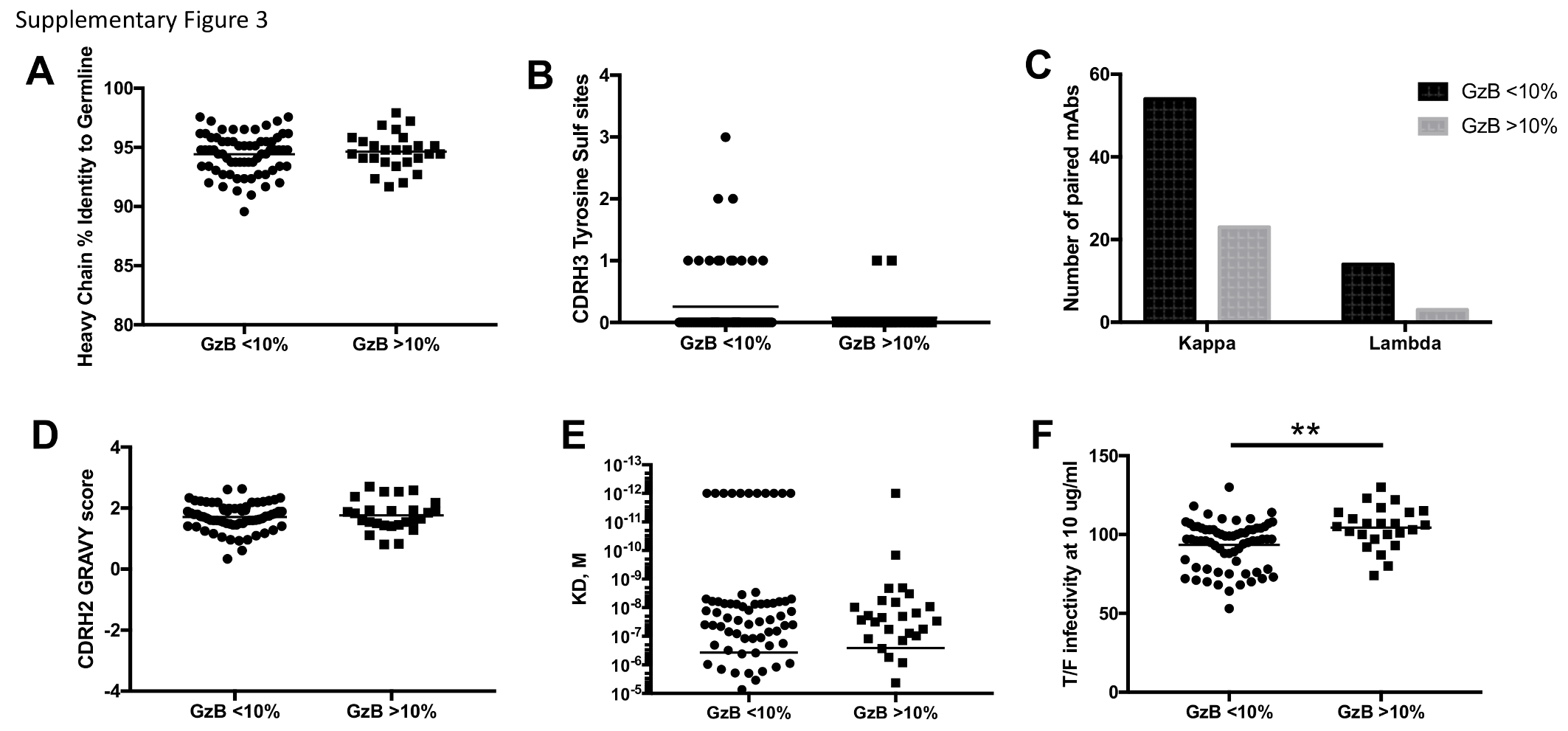

Supplement: Supplementary Figure 3 — Genetic and functional comparison of VH1-69 low ADCC mAbs vs. high ADCC mAbs. VH1-69 mAbs were stratified into two groups, based on low (peak GzB percentage <10%) or high (peak GzB percentage >10%) ADCC activity. No significant differences emerged between these two groups in a number of metrics: (A) heavy-chain percent nucleotide identity to germline (Mann–Whitney, p = 0.74); (B) the number of tyrosine sulfation sites found within the heavy-chain complementarity-determining region-3 (CDRH3) (Mann–Whitney, p = 0.18) (30); (C) kappa or lambda light-chain pairing (Fisher's exact test, p = 0.38); (D) heavy-chain complementarity-determining region-2 (CDRH2) grand average hydropathy (GRAVY) scores (Mann–Whitney, p = 0.89); (E) binding affinity for autologous T/F gp120 (Mann–Whitney, p = 0.76). (F) There was a significant difference between the two groups and their ability to neutralize virions pseudotyped with the autologous T/F Env (Mann–Whitney, p = 0.0015). **p ≤ 0.01. [file Image_3.TIF]

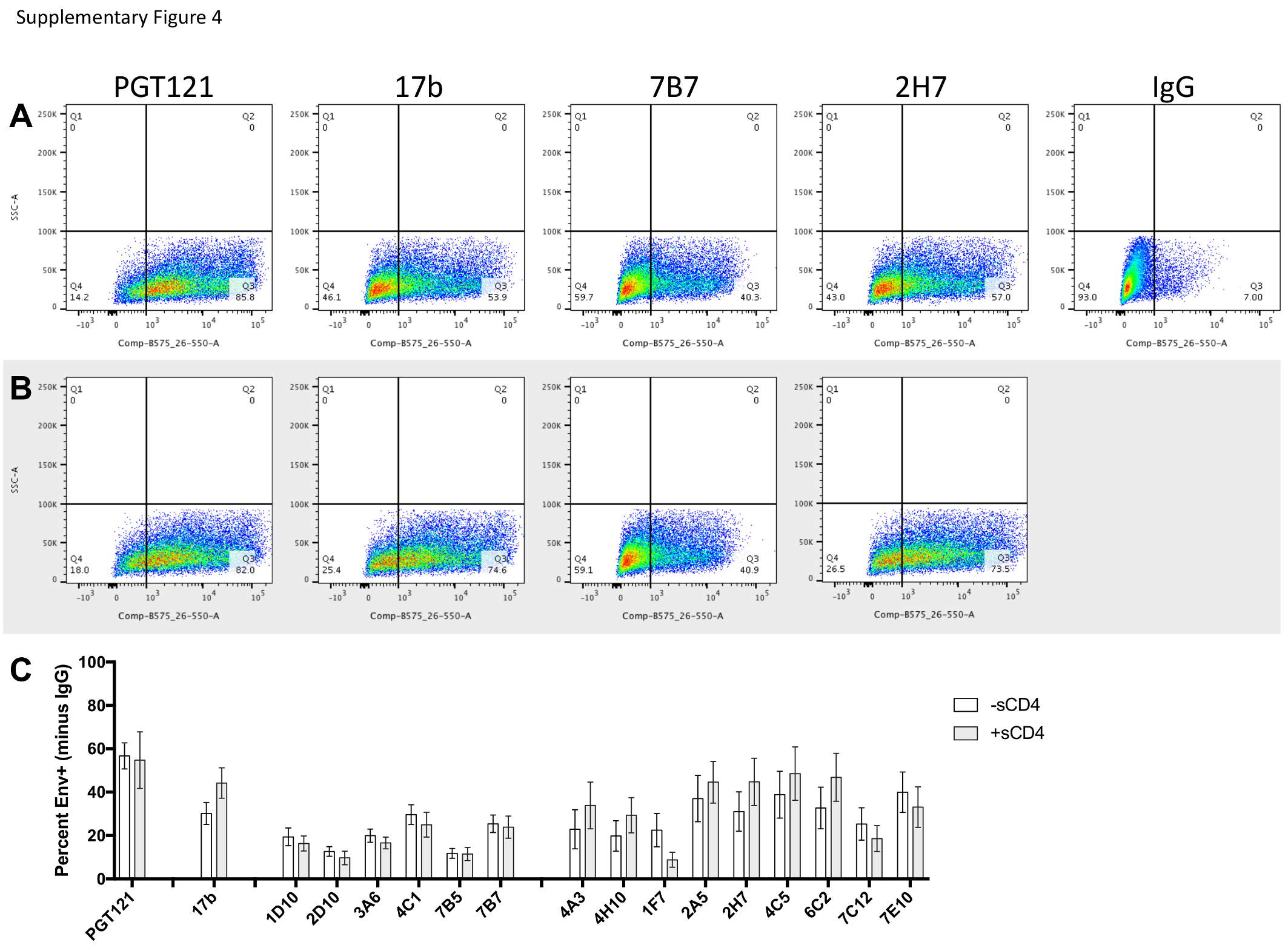

Supplement: Supplementary Figure 4 — Antibody recognition of R66M T/F Env transfected cells in the absence and presence of sCD4. 293T cells were transfected with R66M T/F Envelope expression plasmid. 48-h post-transfection, cells were stained with control and R66M VH1-69 mAbs (indicated above plots), either (A) without or (B) with pre-incubation with sCD4. X-axis indicates PE intensity, Y-axis indicates side scatter area (SSC-A). “Env-positive” cells are found in the lower right quadrant (Q3), percentage of total events per quadrant are indicated. (C) Percent Env-positive cells, minus IgG background, for each mAb in the absence (white) and presence of sCD4 (gray). Columns indicate mean of a minimum of three independent experiments, bars represent SEM. [file Image_4.TIF]
